# Neuronal extracellular vesicles influence the expression, degradation and oligomeric state of fructose 1,6-bisphosphatase 2 in astrocytes affecting their glycolytic capacity

**DOI:** 10.1038/s41598-024-71560-7

**Published:** 2024-09-09

**Authors:** Daria Hajka, Bartosz Budziak, Dariusz Rakus, Agnieszka Gizak

**Affiliations:** 1 Department of Molecular Physiology and Neurobiology, University of Wrocław, 50-335 Wrocław, Poland; 2 Present Address: Łukasiewicz Research Network - PORT Polish Center for Technology Development, 54-006 Wrocław, Poland

**Keywords:** Fructose 1,6-bisphosphatase, Extracellular vesicles, Astrocytes, Neurons, Crosstalk, Glycolysis, Cell biology, Physiology

## Abstract

Fructose 1,6-bisphosphatase 2 (Fbp2) is a regulatory enzyme of gluco- and glyconeogenesis which, in the course of evolution, acquired non-catalytic functions. Fbp2 promotes cell survival during calcium stress, regulates glycolysis via inhibition of Hif-1α activity, and is indispensable for the formation of long-term potentiation in hippocampus. In hippocampal astrocytes, the amount of Fbp2 protein is reduced by signals delivered in neuronal extracellular vesicles (NEVs) through an unknown mechanism. The physiological role of Fbp2 (determined by its subcellular localization/interactions) depends on its oligomeric state and thus, we asked whether the cargo of NEVs is sufficient to change also the ratio of Fbp2 dimer/tetramer and, consequently, influence astrocyte basal metabolism. We found that the NEVs cargo reduced the Fbp2 mRNA level, stimulated the enzyme degradation and affected the cellular titers of different oligomeric forms of Fbp2. This was accompanied with increased glucose uptake and lactate release by astrocytes. Our results revealed that neuronal signals delivered to astrocytes in NEVs provide the necessary balance between enzymatic and non-enzymatic functions of Fbp2, influencing not only its amount but also subcellular localization. This may allow for the metabolic adjustments and ensure protection of mitochondrial membrane potential during the neuronal activity-related increase in astrocytic [Ca^2+^].

## Introduction

The brain is the most energy-consuming organ and in humans, in resting conditions, about 20% of the total glucose-derived energy is dedicated to maintaining its functions^1,2^. Increased neuronal activity is associated with elevation of glucose uptake from the blood and its glycolytic oxidation to lactate by astrocytes^3^. The lactate is then transported to neurons where it supports the mechanisms of memory formation (Long-Term Potentiation—LTP, and Long-Term Depression—LTD) and other processes^4–9^. The mechanism by which the astrocyte-derived lactate stimulates LTP and LTD is not fully understood but lactate oxidation-associated changes in the NADH/NAD^+^ ratio in neurons can influence a set of proteins indispensable for memory formation. They can increase the permeability of the N-methyl-D-aspartate receptor and the activation of calcium/calmodulin-dependent protein kinase 2 by fructose 1,6-bisphosphatase 2 (Fbp2)^10,11^. In turn, neurons enhance capacity of astrocytes to release lactate by stimulating the expression of glucose metabolism proteins^12,13^ in astrocytes.

The mechanism by which neurons affect the glycolytic proteome of astrocytes depends both on the physical interactions of the cells^12^ and on the release of neuronal factors^14^ to the extracellular space, which elevate astrocytic glycolysis, probably through stimulation of cAMP/CREB signaling^13,14^.

Recently, we have demonstrated that extracellular vesicles (EVs) released by astrocytes and neurons affect the expression of Fbp2^15^. The astrocyte-derived EVs elevate the level of neuronal Fbp2 and thus, alter the ability of neurons to form LTP^11^, while neuronal extracellular vesicles (NEVs) reduce the titer of Fbp2 protein in astrocytes through an unknown process. Fbp2 is a ubiquitously expressed enzyme of glycogen synthesis from non-carbohydrates, such as lactate. However, Fbp2 has also numerous non-enzymatic functions. In addition to the effect on LTP, Fbp2, for example, reduces the level of hypoxia-inducible factor 1α (Hif1α) protein in lung cancer^16^ and affects mitochondrial membrane potential in various cell types^17^.

Since these functions are related to subcellular localization (nuclear vs mitochondrial) of Fbp which in turn, depends on the oligomeric state of the enzyme, we asked whether the NEVs-induced reduction of the Fbp2 protein level in astrocytes would correlate with a change in the Fbp2 dimer-tetramer ratio, and we sought to investigate the potential effects of such a change on the basal metabolism of astrocytes.

Here, we present a line of evidence that EVs derived from mouse hippocampal neurons convey factors that decrease the Fbp2 protein level in astrocytes by reduction of Fbp2 transcript and stimulation of the enzyme degradation. These factors also influence the dimer-tetramer ratio of Fbp2 protein. This may allow, on the one hand, to increase the production of lactate by astrocytes, and on the other hand, it may ensure the proper polarization of the mitochondrial membrane during the neuronal activity-related increase in astrocytic [Ca^2+^].

## Results

### NEVs cargo reduces Fbp2 mRNA expression in astrocytes

First, we asked whether the NEVs-dependent reduction of Fbp2 protein level in astrocytes resulted from changes in Fbp2 mRNA level or Fbp2 protein degradation rate.

**Fig. 1:**
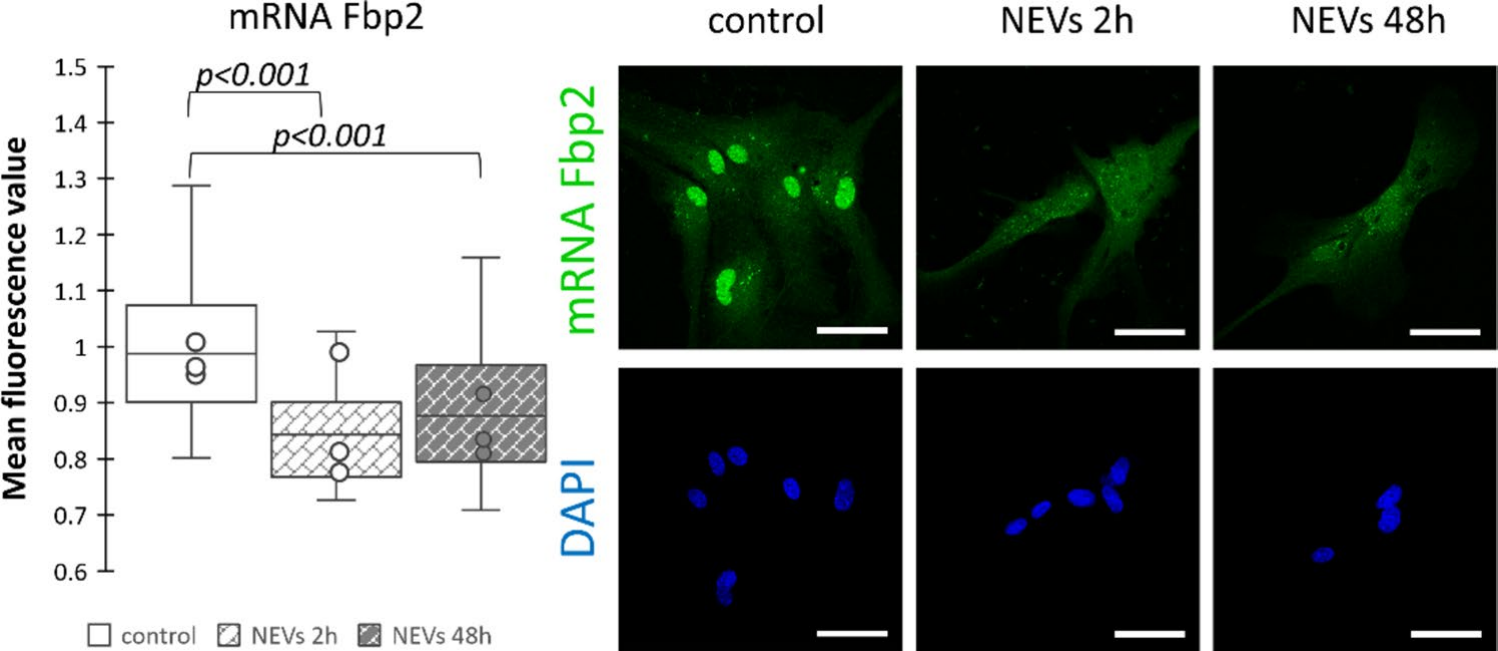
The cargo of neuronal extracellular vesicles reduces Fbp2 mRNA synthesis in astrocytes. Representative images of the FISH experiment and quantification of the fluorescent signal related to Fbp2 mRNA normalized to the control (untreated astrocytes). In a box plot, the horizontal line represents the median for all analyzed cells and dots represent median value for each biological replicate. The experiment was performed in triplicate (N = 3). NEVs—astrocytes treated with neuronal extracellular vesicles. Scale bar = 40 µm. Detailed information on the number of biological replicates, analyzed images or measurements for each condition, and statistical test results for all experiments are provided in Supplementary Table S1.

Fluorescence in situ hybridization (FISH) revealed that the effect of NEVs on Fbp2 mRNA level was almost immediate, and we could see its significant (*p* < 0.01) reduction after 2 h of incubation with NEVs (Fig. 1). The reduction was maintained throughout the duration of the experiment (i.e., 48 h; Fig. 1), and was the highest in astrocytes’ nuclei which suggested that it resulted from the reduction of new Fbp2 mRNA synthesis rather than the stimulation of mRNA degradation.

### NEVs cargo reduces Fbp2 protein level in astrocytes via the Pi3k/Akt pathway

**Fig. 2:**
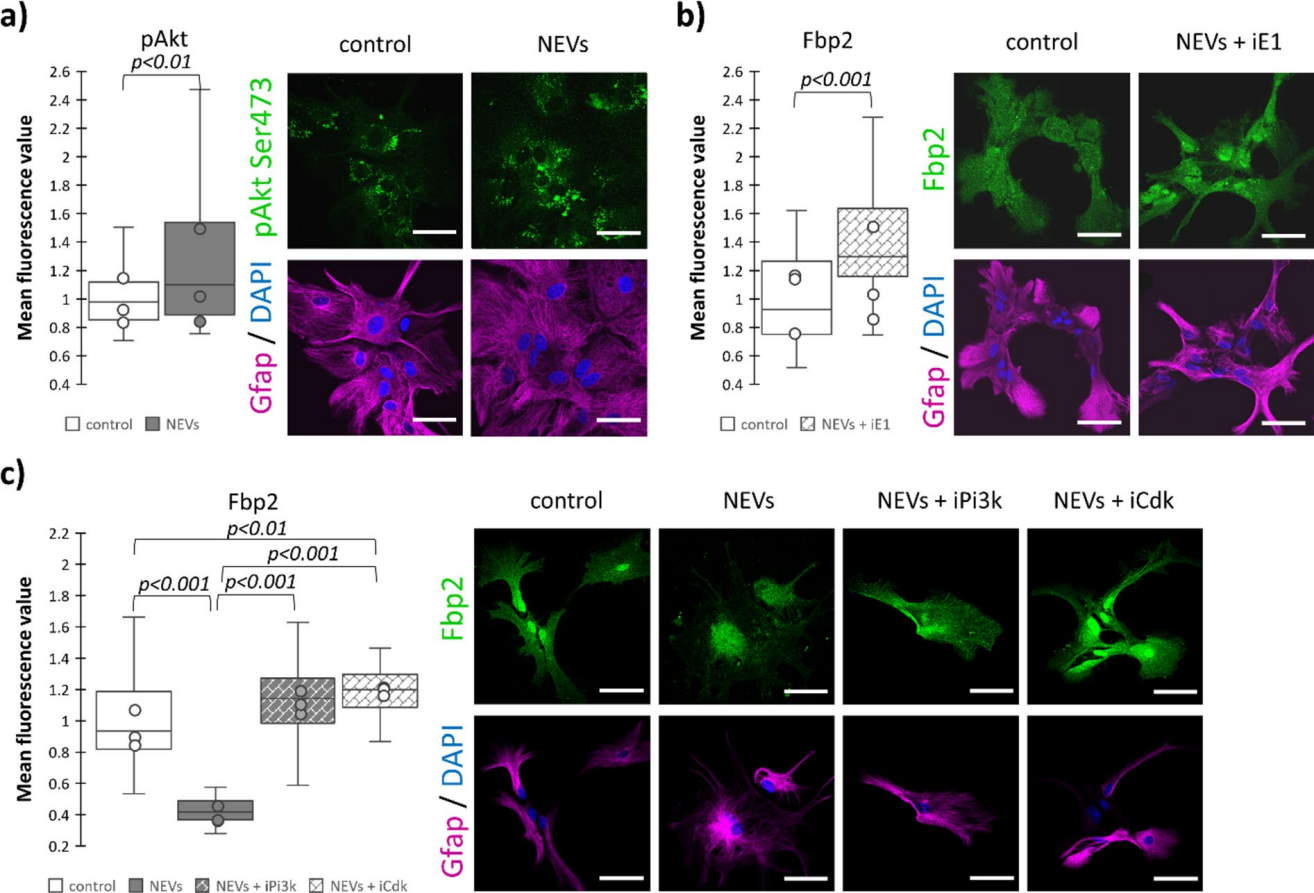
Neuronal extracellular vesicles direct Fbp2 to proteasomal degradation by Pi3k/Akt pathway in astrocytes. Representative confocal images and quantification of the fluorescent signal related to antibodies directed against a given protein (pAkt (**a**); Fbp2 (**b**,**c**)) normalized to a respective control (untreated astrocytes). In a box plot, the horizontal line represents the median for all analyzed cells and dots represent median value for each biological replicate. Each experiment was performed in triplicate (N = 3). Astrocytes were incubated with neuronal extracellular vesicles (NEVs) in the presence of inhibitor of Pi3k (iPi3k; wortmannin), inhibitor of cyclin-dependent kinases (iCdk; roscovitine) or inhibitor of ubiquitin-activating enzyme E1 (iE1; PYR-41). pAkt—Akt kinase phosphorylated on Ser473. Scale bar = 40 µm. Detailed information on the number of biological replicates, analyzed images or measurements for each condition, and statistical test results for all experiments are provided in Supplementary Table S1.

The decrease in mRNA could not be the only mechanism responsible for almost halving the amount of Fbp2 protein in the NEVs-treated astrocytes (^15^, Fig. 2c). The liver isozyme of fructose 1,6-bisphosphatase (Fbp1) can be regulated by activation of the phosphoinositide-3-kinase/Akt kinase (Pi3k/Akt) pathway leading to cyclin-dependent kinase 2 (Cdk2)-dependent phosphorylation of the Fbp1 Ser271 residue and proteasomal degradation of the protein^18^. The Ser271 residue is present also in all mammalian Fbp2. Therefore, we checked if the NEVs-delivered factors activated similar sequence of events. Incubation of astrocytes with NEVs resulted in a significant (*p* < 0.01) increase of the fluorescence related to antibodies directed towards activated (phosphorylated on Ser473) Akt compared to control conditions (Fig. 2a). In turn, the Pi3k inhibitor wortmannin or the Cdk inhibitor roscovitine abolished the NEVs-induced decrease of the intensity of Fbp2 protein-related fluorescence (Fig. 2c). In fact, after inhibition of Pi3k or Cdk2 we observed a significantly higher (23% or 30%, respectively; *p* < 0.01) levels of Fbp2-related fluorescence than in the control cells (not treated with NEVs + inhibitors). Finally, inhibition of the ubiquitination process by application of the ubiquitin-activating enzyme E1 inhibitor PYR-41 increased the intensity of Fbp2-related fluorescence in astrocytes incubated with NEVs by more than 40% (Fig. 2b).

In summary, in astrocytes, Pi3k/Akt/Cdk signaling is a mechanism by which NEVs reduce the Fbp2 protein level. The higher level of Fbp2 protein after inhibition of Pi3K/Akt/Cdk in the NEVs-treated cells compared to the control cells suggests that this pathway is permanently active in astrocytes.

### A certain level of Fbp2 expression is necessary to protect mitochondria

Fbp2 is a regulatory enzyme of synthesis of glucose/glycogen from non-carbohydrate precursors (e.g., lactate). Moreover, Fbp2 reduces glycolytic capacity of cells in a non-catalytic manner. Since astrocytes are supposed to support neurons metabolically with lactate, a high activity of Fbp2 in astrocytes in the presence of neurons would be unfavorable and suppression of the Fbp2 expression would be functionally justified. However, the NEVs-transported signals reduced the total pool of Fbp2 protein by only about half^15^. This suggests that the remaining Fbp2 plays another role in astrocytes.

Fbp2 protects mitochondria (e.g., by stabilizing their membrane potential) against stress signaling such as elevated calcium levels^19,20^ associated to accelerated glycolysis^21^. Therefore, we investigated whether NEVs could affect the Fbp2-mitochondria interaction.

**Fig. 3:**
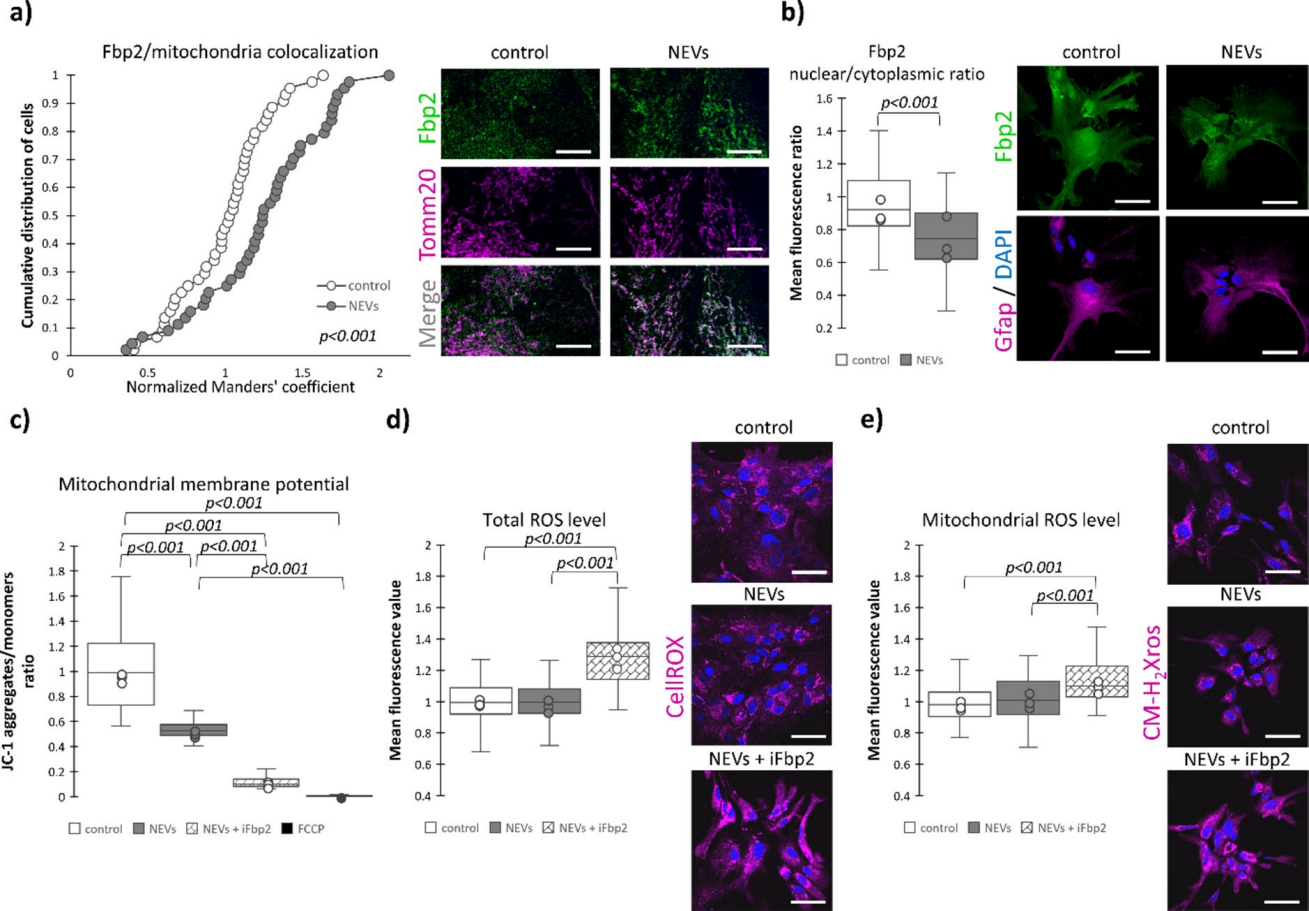
Neuronal extracellular vesicles influence subcellular localization of Fbp2 increasing its interactions with mitochondria. Disruption of Fbp2-mitochondria interaction increases ROS production in the presence of NEVs. (**a**) NEVs treatment increases accumulation of Fbp2 on mitochondria. Representative confocal images and quantification of the Manders’ coefficient M (the percentage of the mitochondria (Tomm20)-related signal overlapped by the Fbp2-related signal) normalized to control. 1 indicates overlap of the fluorescent signals in control conditions, and values above 1 indicate increasing overlap of these signals. Scale bar = 8 µm. (**b**) NEVs treatment decreases Fbp2 nuclear/cytoplasmic ratio. (**c**) Quantification of the red to green fluorescent signal ratio of the JC-1 dye, normalized to the control group. (**d**) Representative confocal images and quantification of the fluorescent signal related to total cellular ROS visualized using the CellROX reagent and (**e**) mitochondrial ROS visualized using CM-H_2_Xros reagent. Scale bar = 40 µm. Control—untreated astrocytes; NEVs—astrocytes treated with neuronal extracellular vesicles; NEVs + iFbp2—astrocytes treated with neuronal extracellular vesicles in the presence of Fbp2 inhibitor/tetramerizing agent-treated astrocytes; FCCP—astrocytes incubated with FCCP, a mitochondria-depolarizing agent. In a box plot, the horizontal line represents the median for all analyzed cells and dots represent median value for each biological replicate. The experiment was performed in triplicate (N = 3). Detailed information on the number of biological replicates, number of analyzed images or measurements for each condition, and statistical test results for all experiments are provided in Supplementary Table S1.

In the NEVs-treated astrocytes, we observed a small (26%) but statistically significant (*p* < 0.001) increase of the percentage of total mitochondria-related fluorescence covered by Fbp2-related fluorescence (Fig. 3a), as determined by the Manders’ overlap coefficient. In other words, although NEVs reduced the total amount of Fbp2 protein in astrocytes, a higher percent of mitochondria interacted with the enzyme. NEVs induced also a significant (*p* < 0.001) decrease in the nucleus/cytoplasm ratio of Fbp2 compared to the untreated astrocytes (Fig. 3b).

Subcellular localization of Fbp2 depends on the oligomeric (dimeric or tetrameric) and conformational (R—active or T—Inactive) state of the protein: only the dimeric^22^ and probably the active (R-state) tetrameric^23^ forms of Fbp2 interact with mitochondria, and the tetrameric Fbp2 is retained in the nucleus^22^. Thus, our observations indicated that NEVs content affected Fbp2 oligomerization and quaternary conformation.

To verify this, we investigated the effect of NEVs on mitochondrial membrane polarization. We used the JC-1 dye, and an uncoupler of mitochondrial membrane potential FCCP as a positive control. Treatment with the uncoupler dissipated the potential (Fig. 3c).

After the incubation of astrocytes with NEVs the mitochondrial membrane polarization decreased (Fig. 3c). This was seemingly unexpected given the observed increase in Fbp2-mitochondria colocalization and the protective effect of Fbp2 on their membrane potential^19,22^. Therefore, we investigated how NEVs would affect this potential if the Fbp2-mitochondria interaction was blocked. We incubated astrocytes with NEVs in the presence of a synthetic compound 5-chloro-2-(N-(2,5-dichlorobenzenesulfonamido))-benzoxazole (abbreviated hereinafter as iFbp2) that induces tetramerization of Fbp2 in its T-state and in consequence, prevents Fbp2-mitochondria interactions^11,23^. As a result, the NEVs treatment induced over three-fold larger decrease of the membrane potential (Fig. 3c). This confirmed the participation of the Fbp2 dimers and/or R-state tetramers in the protection of mitochondrial membrane potential under the NEVs-induced changes.

Furthermore, the NEVs-induced decrease in mitochondria membrane potential did not correlate with an increase in either mitochondrial or total cellular ROS levels in astrocytes (Fig. 3d,e), suggesting that the depolarization was physiological rather than pathological. In turn, the iFbp2-induced tetramerization of Fbp2 caused a significant (*p* < 0.001) elevation of the ROS levels in the presence of NEVs.

Together, these results suggest that in astrocytes, NEVs cargo induced certain processes resulting in mitochondrial membrane depolarization, but the concomitant shift of the Fbp2 dimer-tetramer ratio toward the dimeric form allowed Fbp2 to interact with mitochondria and maintain this depolarization within the non-pathological range.

### Fbp2 protects mitochondria against NEVs-induced [Ca^2+^] increase

Next we tried to find the cause of the NEVs-induced mitochondrial membrane depolarization. Fbp2-mitochondria interaction intensifies during cellular [Ca^2+^] increase^11,19,20^. Calcium ions taken up by mitochondria during physiological processes cause depolarization of the mitochondrial membrane^24,25^.

**Fig. 4:**
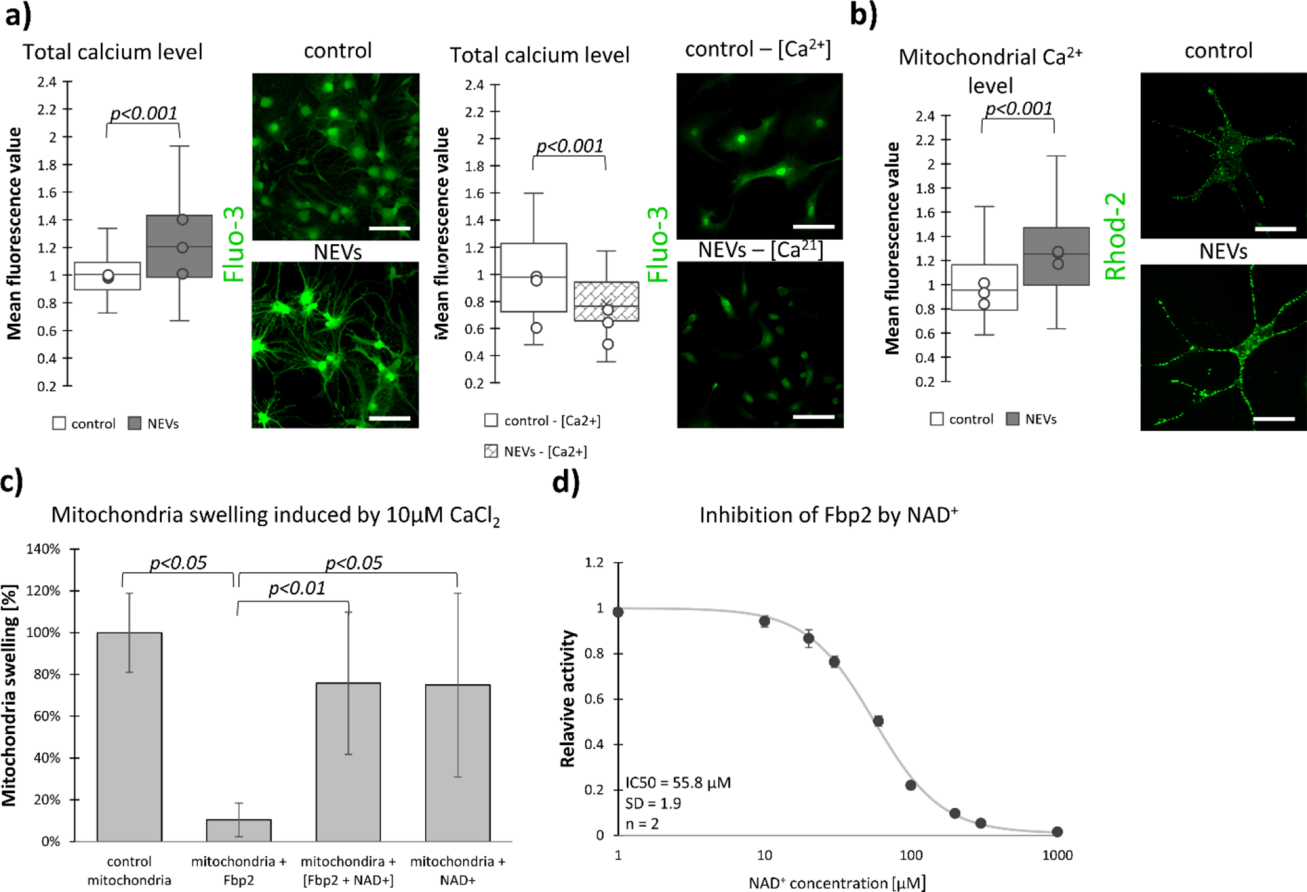
Neuronal extracellular vesicles increase [Ca^2+^] in astrocytes. Fbp2 protects mitochondria against excessive Ca^2+^-induced swelling but saturation of Fbp2 with NAD^+^ abolishes its protective ability. (**a**,**b**) Representative confocal images and quantification of the fluorescent signal related to Fluo-3 (a total [Ca^2+^] indicator) and Rhod-2 (a mitochondrial [Ca^2+^] indicator) normalized to a respective control (untreated astrocytes). The Rhod-2 fluorescence was pseudocolored green. (**c**) The ability of Ca^2+^ to induce swelling of the isolated mitochondria was measured in the untreated mitochondria (control) and in the organelles preincubated with Fbp2, Fbp2 saturated with NAD^+^ ([Fbp2 + NAD +]) or NAD^+^ alone. (**d**) In the presence of 100 µM NAD^+^, majority of Fbp2 molecules should be saturated with this compound. IC50—concentration of NAD^+^ that inhibits Fbp2 activity by 50%, n—Hill constant, SD—standard deviation. In a box plot, the horizontal line represents the median for all analyzed cells and dots represent median value for each biological replicate. Each experiment was performed in triplicate (N = 3). NEVs—astrocytes incubated with neuronal extracellular vesicles; NEVs—[Ca^2+^]—astrocytes incubate with neuronal extracellular vesicles in calcium free media. Scale bar = 80 µm. Detailed information on the number of biological replicates, analyzed images or measurements for each condition, and statistical test results for all experiments are provided in Supplementary Table S1.

In the NEVs-treated astrocytes, we found 22% increase of the total cellular [Ca^2+^], as measured by the Fluo-3 AM fluorescence intensity, compared to the untreated cells (Fig. 4a). This increase was abolished when the cells were incubated in the Ca^2+^-free culture medium (Fig. 4a), which suggested the extracellular origin of the ion. The total [Ca^2+^] rise was accompanied by a similar (27%) NEVs-induced increase of [Ca^2+^] in mitochondria (Rhod-2 AM fluorescence measurement; Fig. 4b), which explained their membrane depolarization.

Thus, the dimeric Fbp2 appeared to be needed to protect mitochondria against excessive depolarization of their membrane in the continuous presence of the depolarization stimulus (i.e., elevated [Ca^2+^]).

Although Ca^2+^ can have beneficial effects on mitochondrial metabolism, Ca^2+^ overload contributes to dysfunction of the organelles^26^. Fbp2 protects isolated mitochondria against Ca^2+^-induced swelling, and this protection is abolished by AMP^20^—an allosteric inhibitor of Fbp2 that tetramerizes the protein. NAD^+^ is another allosteric inhibitor of Fbp2 and thus, a potential T-state tetramer-inducing agent, but its effect on the ability of Fbp2 to reduce mitochondrial swelling remained untested.

We used the spectrophotometric method^20^ to check the effect of NAD^+^ on the Fbp2-dependent reduction of mitochondrial swelling.

Isolated astrocytic mitochondria were suspended in the swelling buffer in the spectrophotometric cuvette, and 10 µM CaCl_2_ was added. This resulted in a decrease in absorbance at 540 nm (the swelling). This decrease was considered as 100% swelling (Fig. 4c). Addition of 100 µM NAD^+^ (the Fbp2-saturating concentration—Fig. 4d) to untreated mitochondria did not influence their volume.

Preincubation of mitochondria with Fbp2 before addition of Ca^2+^ markedly reduced changes in their volume (Fig. 4c), in line with previous results^19,20^. However, when mitochondria were preincubated with NAD^+^-saturated Fbp2, the ability of this protein to protect against the Ca^2+^-induced swelling was abolished.

This demonstrated that NAD^+^-induced Fbp2-tetramerization precludes the enzyme binding to mitochondria, and that the binding is necessary to protect mitochondria against the NEVs-induced cellular [Ca^2+^] increase.

Notably, the titer of NAD^+^ (NAD^+^/NADH ratio) is reduced by activation of glycolysis^27^ and hence, the ability of Fbp2 to protect mitochondria should be higher under such conditions.

### The mechanism of NEVs-induced Fbp2 association with mitochondria

**Fig. 5:**
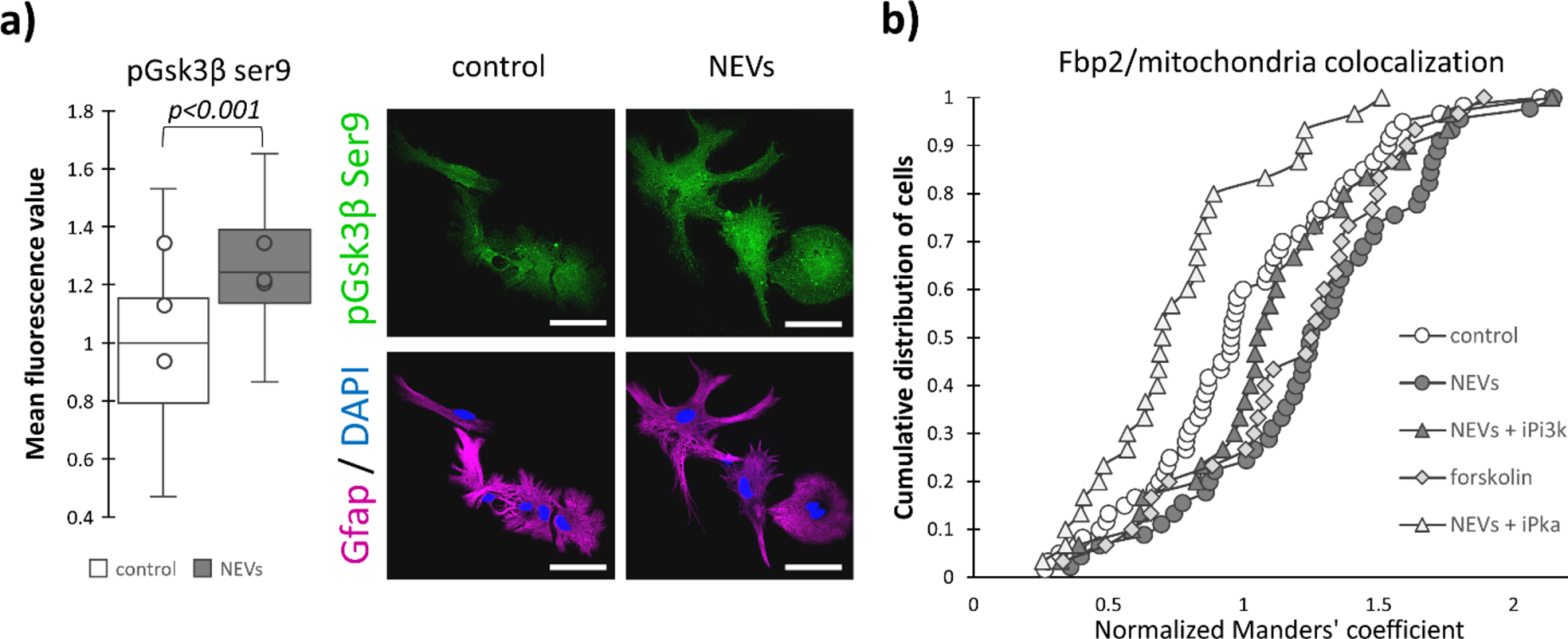
Neuronal extracellular vesicles influence Pi3k/Akt/Gsk3β and Pka pathways to regulate Fbp2 binding to mitochondria. (**a**) Representative confocal images and quantification of the fluorescent signal related to the Gsk3β inhibitory phospholylation. Scale bar = 40 µm. (**b**) Fbp2–mitochondria co‐localization (expressed in Manders’ coefficient normalized to appropriate controls, see section "NEVs increase astrocytic glycolytic capacity of astrocytes") after inhibition or activation of the pathways. 1 indicates overlap of the fluorescent signals in control conditions, values above 1 indicate increasing and below 1 indicate decreasing overlap of these signals. Astrocytes were incubated with: NEVs—neuronal extracellular vesicles; iPi3k—inhibitor of Pi3k (wortmannin); forskolin—activator of Pka; iPka—inhibitor of Pka (KT5720). Control—untreated astrocytes. In a box plot, the horizontal line represents the median for all analyzed cells and dots represent median value for each biological replicate. Each experiment was performed in triplicate (N = 3). Detailed information on the number of biological replicates, analyzed images or measurements for each condition, and statistical test results for all experiments are provided in Supplementary Table S1.

Then we sought for the molecular mechanism of the NEVs-induced association of Fbp2 with astrocyte mitochondria. Activation of the Pi3k-Akt pathway by NEVs (Fig. 2) should result in the inhibition (phosphorylation on Ser9) of glycogen synthase kinase 3β (Gsk3β). Inhibition of the kinase stimulates Fbp2-mitochondria interaction in other cell types^19^. Here, we found that in astrocytes, NEVs induced a significant (~ 25%, *p* < 0.001) increase in the fluorescence related to the Ser9-phosphorylated Gsk3β (Fig. 5a).

Determination of the Manders’ coefficient revealed that inhibition of Pi3k reduced the ability of NEVs to increase FBP2-mitochondria co-localization (Fig. 5b; control vs NEVs *p* < 0.001, D = 0.38; control vs NEVs + iPi3k *p* = 0.073, D = 0.28), which suggested that in astrocytes, activation of a canonical pathway leading to inhibitory phosphorylation of Gsk3β regulated the Fbp2-mitochondria interaction.

Another universal mechanism leading to inhibitory phosphorylation of Gsk3β is mediated by Pka^28^. Preincubation of astrocytes with the Pka inhibitor (KT5720) abolished the ability of NEVs to increase Fbp2-mitochondria interaction (Fig. 5b; NEVs vs NEVs + iPka *p* < 0.001 D = 0.6), and even decreased this interaction compared to control conditions (Fig. 5b; control vs NEVs + iPka *p* < 0.01, D = 0.38). In turn, incubation of control astrocytes with forskolin (which stimulates adenylate cyclase to produce cAMP, a Pka activator) increased the Manders’ coefficient similarly to the NEVs treatment (NEVs vs forskolin *p* = 0.58, D = 0.18), and clearly showed that forskolin induced Fbp2-mitochondria interaction (Fig. 5b; control vs forskolin *p* < 0.01, D = 0.37) in astrocytes.

Inhibition of both Pka and Pi3k prevented NEVs from increasing the level of Gsk3β phosphorylation at Ser9 (Supplementary Fig. S1).

Additionally, analogously to Pi3k inhibition, inhibition of Pka prevented NEVs from reducing the amount of Fbp2 protein in astrocytes (Supplementary Fig. S2). In turn, incubation of the cells with forskolin slightly, but significantly (*p* < 0.01) reduced Fbp2 amount (Supplementary Fig. S2).

Together, these results suggest that Pi3k/Akt/Gsk3 and Pka pathways regulate Fbp2 protein fate in the NEVs-treated astrocytes.

### NEVs increase astrocytic glycolytic capacity of astrocytes

One of the Fbp2 non-enzymatic functions is the reduction of hypoxia-inducible factor 1α (Hif1α) protein level in cancer cells^16^. Hif1α increases transcription of genes encoding glycolytic enzymes, and glucose and lactate transporters^29^. In turn, glycolysis activation reduces the NAD^+^ titer (NAD^+^/NADH ratio)^27^ and hence, increases the ability of Fbp2 to protect mitochondria (see “Section ROS, [Ca^2+^] and mitochondria polarization assays”). Moreover, we have previously shown that the Hif1α protein-related fluorescence in astrocytes co-cultured with neurons was about 20% higher than in the monoculture^15^. Therefore, we tested the influence of NEVs on glycolysis in astrocytes using immunofluorescence (IF). If the observed changes in fluorescence were lower than 20%, we confirmed the obtained results with the Western blot (WB) technique.

**Fig. 6:**
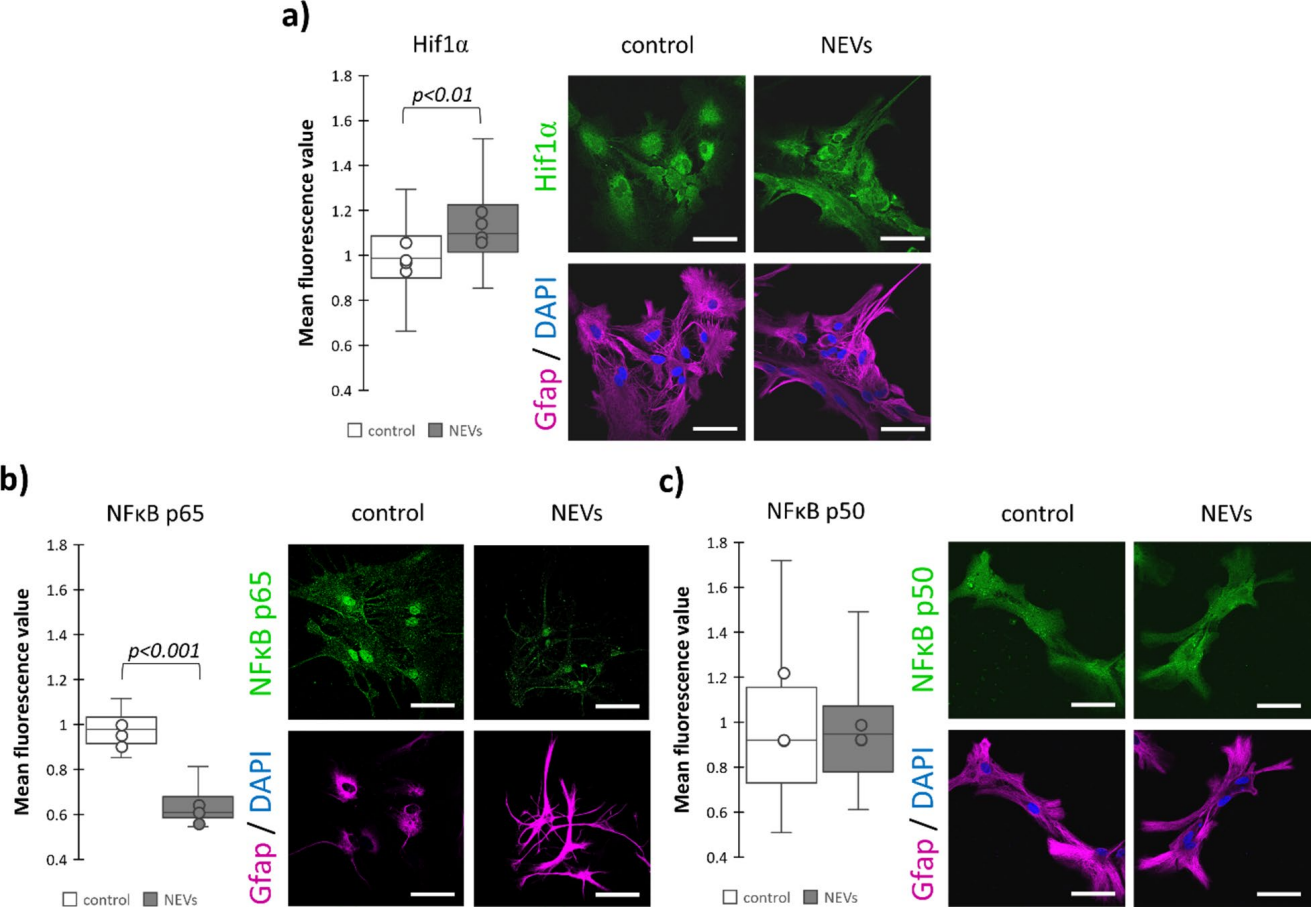
Neuronal extracellular vesicles increase the Hif1α and reduce the p65 but not p50 subunit of NFκB amounts in astrocytes. Representative confocal images and quantification of the fluorescent signal related to antibodies directed against a given protein normalized to a respective control (untreated astrocytes). In a box plot, the horizontal line represents the median for all analyzed cells and dots represent median value for each biological replicate. Each experiment was performed at least in triplicate ((**a**) N = 4, (**b**) N = 3, (**c**) N = 3). NEVs—astrocytes treated with neuronal extracellular vesicles-treated astrocytes; Hif1α—hypoxia-inducible factor 1α; NFκB—nuclear factor kappa-light-chain-enhancer of activated B cells. Scale bar = 40 µm. Detailed information on the number of biological replicates, analyzed images or measurements for each condition, and statistical test results for all experiments are provided in Supplementary Table S1.

Incubation of astrocytes with NEVs resulted in a small (IF: 11%, WB: 10%), but significant (*p* < 0.01) increase of Hif1α protein level (Fig. 6a; Supplementary Fig. S3).

In turn, the level of NfκB (nuclear factor kappa-light-chain-enhancer of activated B cells) p65 (RelA) subunit was markedly (about 40%) reduced by NEVs (Fig. 6b). In healthy mouse cells, basal activity of this subunit restricts glycolytic activity and lactate production, and its inhibition causes cellular reprogramming to aerobic glycolysis^30^. The amount of the p50 subunit remained unchanged by NEVs (Fig. 6c).

**Fig. 7:**
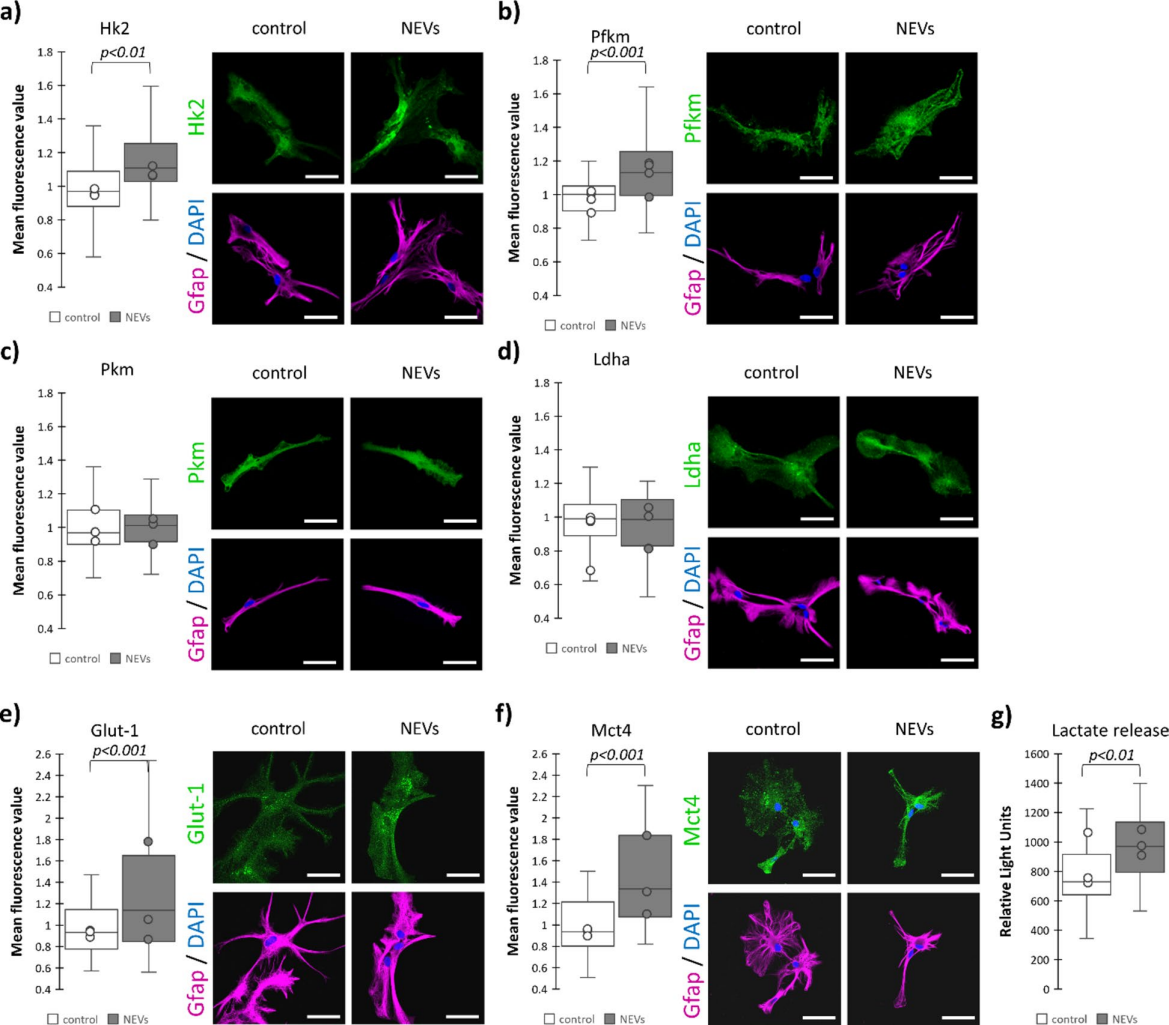
Neuronal extracellular vesicles influence glycolytic capacity and increase lactate release from astrocytes by elevating the expression of hexokinase, phosphofructokinase, and glucose and monocarboxylate transporters. (**a**)–(**f**) Representative confocal images and quantification of the fluorescent signal related to antibodies directed against a given protein normalized to a respective control (untreated astrocytes). (**g**) Quantification of lactate release to extracellular space by astrocytes. In a box plot, the horizontal line represents the median for all analyzed cells and dots represent median value for each biological replicate. Each experiment was performed at least in triplicate (a): N = 3, b): N = 4, c): N = 3, d): N = 4, e) = 3, f) = 3,  g) = 3). NEVs—astrocytes treated with neuronal extracellular vesicles; Hk2—hexokinase 2; Pfkm—phosphofructokinase M; Pkm—pyruvate kinase type M; Ldha—lactate dehydrogenase A, Glut-1—glucose transporter 1; Mct4—monocarboxylate transporter 4. Scale bar = 40 µm. Specific information on the number of biological replicates, analyzed images or measurements for each condition, and statistical test results for all experiments are provided in Supplementary Table S1.

Moreover, NEVs induced a slight but significant increase in the rate-limiting enzyme of glycolysis, hexokinase 2 (Hk2; ICC: 14%, *p* < 0.01; WB: 21%) and in a regulatory enzyme of the pathway, the M isoform of phosphofructokinase (Pfkm; ICC: 13%, *p* < 0.001; WB: 31%) protein levels (Fig. 7a,b; Supplementary Fig. S3). We did not observe changes in another regulator of glycolysis—pyruvate kinase type M (Pkm), and lactate dehydrogenase A (Ldha) levels (Fig. 7c,d; Supplementary Fig. S3), but because of the high abundance of these proteins in the cell, their activity is regulated by posttranslational modifications/allosteric effectors and the substrate concentration rather than on the expression level^31–33^.

NEVs induced also 23% elevation in IF signal related to a glucose transporter Glut-1 (Fig. 7e) and 43% increase in the protein level of monocarboxylate transporter Mct4 (Fig. 7f), responsible for lactate secretion by astrocytes^34^ and capable of exporting this monocarboxylate in a high-lactate microenvironment^35^. In line with these results, the luminometric measurement indicated 33% increase of the amount of lactate released to the culture medium (Fig. 7g).

## Discussion

Astrocytes modulate numerous aspects of the brain homeostasis. In the process called astrocyte-neuron cross-talk, the glial cells influence neuronal physiology and vice versa^36^. The cross-talk significantly elevates the levels of regulatory proteins of glycolysis/glycogen metabolism in astrocytes^12^, and the lactate formed in this process can be used by neurons to produce energy in oxidative phosphorylation and can stimulate memory formation in a non-metabolic manner^4,37^. One of the means of intercellular communication is the secretion of extracellular vesicles (EVs) into the extracellular space^38^.

In hippocampal neurons and astrocytes, the EVs-dependent cross-talk reciprocally regulates the amount of Fbp2 protein in these cells. In astrocytes, this crosstalk results in about 40% reduction of Fbp2 protein abundance^15^. Fbp2 enzymatic activity promotes glycogen synthesis, e.g. from lactate, while reducing the glycolytic flux. Fbp2 is also a pro-survival and pro-memory-formation protein^11,19^. Its multifunctionality is a result of the existence of different oligomeric forms of Fbp2 (dimers and tetramers in various conformational states) in the cell, capable of interacting with distinct proteins/subcellular structures^22^.

Therefore, we asked how the factors transported to astrocytes in NEVs reduce Fbp2 protein levels and whether they are sufficient to also cause a change in the quaternary form of Fbp2 and, consequently, influence the basal metabolism of astrocytes.

The obtained results demonstrated that the NEVs cargo quickly reduced Fbp2 mRNA in astrocytes, which could be due to the blockade of its synthesis and/or the stimulation of its degradation, e.g. by miRNA delivered by the vesicles^39^. This reduction may seem unexpected because neuronal conditioned medium (NCM) had no such effect^15^. However, our previous observation might have resulted from different methodology of NCM and NEVs collection. Since cultured neurons do not survive the complete exchange of the culture medium, only a part of the medium was collected from a well and was used to stimulate astrocytes in the previous experiment with the NCM. In contrast, since NEVs constitute only a small fraction of the NCM, a whole portion of the medium had to be collected for their efficient isolation. The NEVs are probably not homogenously distributed in the volume of the medium (they adhere to the cells) and the titer of NEVs in the previously used NCM could be significantly lower than in the experiments with purified NEVs.

Our results also showed that NEVs cargo can influence the amount of Fbp2 protein in astrocytes by increasing its proteasomal degradation via activation of the Pi3k/Akt/Cdk pathway. This direct mechanism of protein amount regulation is consistent with previous observations that Fbp2 titer reduction in astrocytes is relatively fast, and that half an hour is sufficient time to observe the EV uptake into cells^40^.

Fbp2 homodimers and homotetramers play different roles in the cell. The dimeric (and, probably, R-tetrameric) form of Fbp2 protects mitochondria against excessive depolarization and thus, reduces apoptosis^11,19^. The tetrameric form of Fbp2 accumulates in cells’ nuclei^22^, which correlates with promotion of cell division^41,42^. Therefore, the subcellular localization of Fbp2 is indicative of its oligomeric/conformational state and also a cellular role.

Treatment of astrocytes with NEVs induced translocation of Fbp2 to mitochondria and reduction of its nucleus/cytoplasm ratio. Thus, the NEVs-delivered factor(s) not only changed the total amount of Fbp2 protein in astrocytes, but also increased its dimer/tetramer ratio and, presumably, stabilized the remaining tetrameric pool of Fbp2 in the active R-state that (just like dimers) can interact with mitochondria.

Thus, we asked about the mechanism and physiological consequences of this subcellular relocation of Fbp2.

In cardiac myocytes, binding of Fbp2 to mitochondria is stimulated by inhibition of Gsk3β induced by the Pi3k/Akt pathway^19^. Here, we showed that the level of inhibitory Gsk3β phosphorylation in astrocytes was increased under the NEVs treatment. However, although NEVs activate Akt kinase, our results indicated that in astrocytes, the Pka-dependent signaling pathway may be the main factor stimulating the Gsk3β inhibition-mediated association of Fbp2 with mitochondria. Pka phosphorylates the Ser9 residue of Gsk3β^43^, and the highest reduction of the mitochondria-bound Fbp2 was observed just after Pka inhibition (Fig. 5b). The broad spectrum of substrates and complex cross-talk between the two kinases may, however, suggest their exchangeable role in the regulation of Gsk3β and thus, Fbp2-mitochondria colocalization, depending on the metabolic state of cells.

Based on our previous findings, we suspected a protective role of Fbp2 towards astrocyte mitochondria (protection against excessive depolarization induced by an increase in [Ca^2+^]^19^), and this was confirmed by several observations. First of all, the NEVs-delivered factors induced increase in cellular and mitochondrial [Ca^2+^]. Since this was not accompanied by an increase in either total or mitochondrial ROS production, it could be assumed that the uptake of the cation was in the physiological range and did not result in the Ca^2+^ overload^26^. In line with the literature data^24,25^, the Ca^2+^ uptake by mitochondria correlated with significant depolarization of their membrane. However, after chemically induced (using iFbp2) transition of the total cellular pool of Fbp2 to its T-state tetramer, the NEVs-induced depolarization was 3 times larger. Moreover, in the presence of the Fbp2-tetramerizing agent, NEVs elicited a significant increase of mitochondrial and total cellular ROS production.

Additionally, in in vitro experiments, purified Fbp2 protected isolated astrocyte mitochondria against Ca^2+^-induced swelling, but this protection was abolished upon saturation of Fbp2 with NAD^+^—an allosteric inhibitor of Fbp2, which stabilizes the enzyme in its T-state. This suggests that NAD^+^, similarly to AMP^19^, could function as physiological agent that precludes binding of the protein to mitochondria, and that the binding is necessary to protect mitochondria against the NEVs-induced cellular [Ca^2+^] increase.

The application of NEVs is supposed to (partially) mimic the presence of neurons in the culture of astrocytes. Thus, the changes in [Ca^2+^] suggested that the NEVs-delivered factors “primed” astrocytes to a process normally activated in the presence of neurons and requiring an elevated [Ca^2+^] in astrocytes. Mitochondrial Ca^2+^ uptake plays a substantial role in regulating [Ca^2+^] since they can both respond to and modify cytoplasmic Ca^2+^ elevations^44^. Thus, the NEVs-induced dimeric/R-tetrameric Fbp2-mitochondria interaction may be an important factor in preparing astrocytes for the presence of neurons and for metabolic cooperation of these cells.

Moreover, a reduction of the NAD^+^ titer (NAD^+^/NADH ratio)^27^ is one of the consequences of glycolysis activation is and hence, the ability of Fbp2 to protect mitochondria should be higher under such conditions.

Fbp2 decreases glycolytic flux by catalyzing the opposite reaction to that catalyzed by phosphofructokinase, a regulatory enzyme of glycolysis. However, in brain, this effect is probably very weak as the titer of Pfk is many times higher than Fbp^45^. Fbp2 downregulates glycolysis also indirectly, by interactions with Hif1α^16^—one of the main regulators of glycolytic enzymes and glucose and monocarboxylate transporters protein expression^46^.

Since Hif1α activity and protein level can be reduced by both fructose 1,6-bisphosphatase isozymes (Fbp1 and Fbp2)^16,47^, we checked if the NEVs-induced decrease of the Fbp2 (the main Fbp isoform in astrocytes^48,49^) protein level was correlated with Hif1α reduction. It has previously been shown that co-culturing of astrocytes with neurons increases ~ 20% the Hif1α protein in astrocytes, as compared to their monoculture^15^. Here, we showed that the NEVs-delivered factors were sufficient to induce statistically significant increase of Hif1α protein level.

In cancer cells, Hif1α promoter is activated by NFκB subunits, mainly p50^50^. In turn, Fbp1–NfκB p65 interaction induces proteasomal degradation of this subunit and inhibits tumor growth^51^. Unlike cancer cells, in normal mouse cells, the p65 subunit of NfκB restricts glycolytic activity and lactate production, and inhibition of this subunit causes reprogramming to aerobic glycolysis^30^. Moreover, aging-associated decrease in the rate of glycolysis correlates with elevation of the p65 titer in rat astrocytes^52^. Here, we observed that the level of p65, but not p50, was markedly reduced by the NEVs-transported factors, in agreement with the observed intensification of glycolysis. It is, however, unclear whether and how the reduction of Fbp2 is directly involved in the decrease of NfκB in the NEVs-treated astrocytes.

Fbp isoforms differ in the arrangement of their subunits within tetrameric quaternary conformations and sensitivity to allosteric inhibitors (AMP, NAD^+^) which drive the conformational changes^53^. Thus, it might be hypothesized that Fbp2 may bind and regulate NfκB in a different manner than Fbp1.

Consistent with the results for Hif1α and NfκB p65, in the presence of NEVs, we observed statistically significant increases in the amounts of crucial glycolytic enzymes (Hk2 and Pfkm) and the transporter involved in the glucose uptake (Glut-1). While the increase in Pfkm is probably not the key factor affecting the maximal rate of glycolysis (Pfkm is a regulatory but not rate-limiting glycolytic enzyme), the elevation of Glut-1 (~ 25%) and the rate-limiting Hk2 (~ 20%) may increase the glycolytic capacity of astrocytes by increasing the availability of substrate for the pathway. Such stimulation was corroborated by over 30% increase of the amount of lactate released to the culture medium and over 40% increase of the amount of Mct4 protein involved in secretion of lactate to the extracellular space. Together, these results suggested that signals contained in NEVs were sufficient to activate in astrocytes the process of metabolic support of neurons. Moreover, we observed that EVs released by astrocytes significantly increased the amount of Mct2 protein, a monocarboxylate transported dedicated to lactate uptake by cells, in neurons (Supplementary Fig. S4), but this part of our research is ongoing.

Supplementary Fig. S5 summarizes the effect of NEVs-delivered cargo on Fbp2, and the consequences of this in astrocytes.

Based on already published and presented here results it could be concluded that there are several populations of Fbp2 in astrocytes, which titers can be regulated by NEVs.

1. It has been shown that nuclear Fbp2 adopts tetrameric conformation because the nuclear export signal is located on those dimer surfaces that are responsible for the tetramer formation, and the dimerization of Fbp2 is prerequisite to its export from the nucleus^22^. NEVs stimulate the export of Fbp2 from nuclei, however, the molecular mechanism and machinery directly involved in the NEVs-induced stabilization of the dimeric form is yet unknown. The results of our research suggest that an important element of this process is the inhibition of Gsk3β.
2. A pool of the dimeric and R-state tetrameric Fbp2 can interact with and protect mitochondria against the NEVs-induced metabolic changes.
3. The free cytosolic dimer and R-state tetramer of Fbp2 can participate in gluconeogenesis, lowering thus the glycolytic rate, but the NEVs-initialized influx of Ca^2+^ can inhibit competitively the enzyme, as the concentration of Ca^2+^ needed to inhibit Fbp2 is about 1 µM^54^.
4. A part of the cytoplasmic pool Fbp2 can be saturated with NAD^+^ and AMP, and adopt the T-state tetrameric conformation, since the concentration of free NAD^+^ in the cytoplasm of cultured cells is 40–70 µM^55^ and AMP is about 20–40 µM^56^. In such conditions, over 99% of the free cytosolic Fbp2 should be tetramerized in the T-state and inactivated by these allosteric inhibitors^57,58^. This would additionally reduce the Fbp2-activity driven gluconeogenesis from lactate.
  The ratio of different Fbp2 conformations within the cell is unknown but the observed NEVs-induced downregulation of Fbp2 level in astrocytes may prevent Fbp2 interaction with and degradation of Hif1α^16^ and/or (supposedly) inhibition of the Hif1α transcriptional activity.

Concluding, the results of our study indicate that neuronal signals delivered to astrocytes in extracellular vesicles, provide the necessary balance between the enzymatic and non-enzymatic functions of Fbp2 in these cells, by influencing both the amount and the dimer-tetramer ratio of Fbp2 protein. This results in the increase of the lactate production by astrocytes and ensures the proper polarization of the mitochondrial membrane during the neuron-induced, astrocyte activity-related increase in [Ca^2+^].

## Methods

### Cell culture and treatment

Animals were obtained from the Experimental Animal Facility of the Medical University of Wrocław. Astrocytes were isolated from hippocampi of newborn BALB/C mice as described previously^12^. The protocol complied with standards of EU Directive 2010/63/EU for animal experiments and was approved by the II Local Scientific Research Ethical Committee, Wroclaw University of Environmental and Life Sciences (permission no WNB.464.2.2020.IR), and the study is reported in accordance with ARRIVE guidelines. To obtain a primary cell culture, hippocampi dissected from animals originated from one litter were pooled together before plating. Usually, for one primary culture 6–8 newborns were used. A biological replicate will refer to independently obtained cell cultures from different litters of mice. Hippocampal dissection was performed on ice in dissection medium (DM; 81.8 mM Na_2_SO_4_, 30 mM K_2_SO_4_, 5.8 mM MgCl_2_, 0.25 mM CaCl_2_, 1 mM HEPES, 0.2 mM NaOH, 20 mM glucose). Dissected hippocampi were rinsed 3 times with DM and incubated with 0.25% trypsin 0.02% EDTA in DM in a 1:2 ratio for 15 min at 37 °C and then in a 2:1 ratio under the same conditions. Then explants were washed with a standard astrocytic culture medium (DMEM with 1g/L glucose, 3.7 g/L sodium carbohydrate, 0.11 g/L sodium pyruvate, 94 mg/L D-valine, supplemented with 10% FBS, 100 U/L penicillin, 0.1 mg/L streptomycin, 2 mM glutamine) to stop trypsin activity. Then the tissue was gently dissociated by pipetting. The suspension was centrifuged 500×*g* for 10 min at room temperature (RT) and the pellet was suspended in a fresh portion of astrocytic culture medium. Cells were counted using Muse Cell Analyzer (Merck KGaA, Darmstadt, Germany) and Muse Count & Viability kit (Merck KGaA, Darmstadt, Germany, LUMIMCH100102), seeded at a density 50,000 cells/cm^2^ into 25 cm^2^ culture flasks, and cultured in 37 °C in a humidified atmosphere with 5% CO_2_ (Forma™ Steri-Cycle™ i160 incubator, ThermoFischer Scientific, Waltham, MA, USA). They were passaged every 8–10 days. All experiments were performed on astrocytes between the 2nd and 4th passage, seeded onto 18 mm slides in a 12-well plate that have previously been coated with a coating solution (borate buffer: 80 mM H_3_BO_3_, 20 mM Na_2_B_4_O_7_; 2.5 µg/mL laminin, 0.1 mg/mL poly-L-lysine) and thoroughly rinsed. Two days before every experiment the astrocytic culture medium was replaced with medium consisting of Neurobasal A (ThermoFisher Scientific, Waltham, MA, USA, A2477501), 2% B27 Supplement (ThermoFisher Scientific, Waltham, MA, USA, 17504044), 0.5 mM glutamine (Merck KGaA, Darmstadt, Germany, G7513), 12.5 μM glutamate (Merck KGaA, Darmstadt, Germany, G1251), 1% penicillin/streptomycin (bio-west, Bourg, France, L0022-100), to minimize the influence of extracellular vesicles present in Fetal Bovine Serum, which is a component of astrocytic medium. In the Neurobasal A-based, medium B27 is used instead of FBS and it does not contain any vesicles. This is also the standard medium used for neuronal monoculture and astrocyte-neuron co-cultures.

In the course of experiments, cultured astrocytes were treated for 48 h with different inhibitors/activators: Pi3k inhibitor—1 μM wortmannin (Merck KGaA, Darmstadt, Germany), Cdk inhibitor—10 μM roscovitine (Merck KGaA, Darmstadt, Germany), adenylyl cyclase activator—10 μM forskolin (Merck KGaA, Darmstadt, Germany), Pka inhibitor—2 μM KT5720 (Merck KGaA, Darmstadt, Germany), ubiquitin-activating enzyme E1 inhibitor—20 μM PYR-41 (Merck KGaA, Darmstadt, Germany) or Fbp2 inhibitor—5 μM 5-chloro-2-(N-(2,5-dichlorobenzenesulfonamido))-benzoxazole (Cayman Chemicals, Ann Arbor, MI, USA)^59^, that tetramerizes the enzyme^11,23^. All the chemicals were added to the culture medium shortly before the addition of neuronal extracellular vesicles.

### Isolation of neuronal extracellular vesicles

Hippocampal neurons were isolated and cultured as described in^60^, with a minor alteration—the concentration of glucose in the culture medium was 2.5 mM. Hippocampi dissection and trypsinization was performed as described above. After trypsinization, explants were washed with warm MEM-FBS (MEM, 10% FBS, 1% MEM Non-Essential Amino Acid Solution, 1% GlutaMAX Solution, 100 U/L penicillin, 0.1 mg/L streptomycin, 20 mM glucose) and dissociated by gentle pipetting. The suspension was centrifuged 500×*g* for 10 min at RT, and the pellet was suspended in a fresh portion of MEM-FBS. Neurons were seeded at a density 25,000 cells/cm^2^ directly onto 18 mm slides placed in a 12-well plate that have previously been coated as described above. 2 h after seeding MEM-FBS was replaced by a neuronal medium (Neurobasal A, 2% B27 Supplement, 0.5 mM glutamine, 12.5 μM glutamate, 1% penicillin/streptomycin). Pure neuronal monocultures were obtained by addition of 2.5 µM Ara-C to the medium. The purity of the monocultures was systematically checked by immunodetection of Map2 (neuronal marker) and Gfap (glial marker).

Neuronal extracellular vesicles (NEVs) were isolated from the conditioned medium of 14–16-day-old monocultures of neurons as described in^61^. The collected medium was centrifuged to remove dead cells and membrane debris: 3000×*g* for 10 min at 4 °C followed by 10,000×*g* for 20 min at 4 °C, in both cases the pellet was discarded. The supernatant was centrifuged 100,000×*g* for 70 min at 4 °C. After the ultracentrifugation the supernatant was removed and the pellet (extracellular vesicles) was suspended in a fresh portion of neuronal medium and applied for 48 h to astrocytic monoculture in 1:1 ratio (i.e. if the NEVs were isolated from 1 ml of neuronal culture medium aspired from one well in a 12-well culture plate, they were re-suspended in 1 ml of the culture medium and applied on astrocytes cultured in one well of a 12-well plate). NEVs isolated from 1 ml of neuronal culture medium contained approximately 42 µg protein and 30 pg miRNA. NEVs were always used for the experiments immediately after isolation. The culture medium used in the experiments contained B27 (ThermoFisher Scientific, Waltham, MA, USA, 17504044) instead of serum, so it did not contain serum-derived vesicles.

### Immunofluorescence

The immunofluorescent staining of paraformaldehyde-fixed cells was performed as described before^12^. The cells were incubated overnight at 4 °C with respective primary antibodies: rabbit anti-hexokinase type II (1:1000, Merck KGaA, Darmstadt, Germany, AB3279), rabbit anti-Pfkm (1:1000, Merck KGaA, Darmstadt, Germany, HPA002117), rabbit anti-Pkm2 (isoform M1) (1:1000, Merck KGaA, Darmstadt, Germany, SAB4200094), rabbit anti-lactate dehydrogenase A (1:1000, bio-techne, Toronto, Canada, NBP1-48336), mouse anti-Glut-1 (1:1000, Abcam, Cambridge, UK, ab40084), rabbit anti-Mct4 (1:1000, Abcam, Cambridge, UK, ab74109), mouse anti-Hif1α (1:1000, Merck KGaA, Darmstadt, Germany, SAB5200017), rabbit anti-NfκB p65 (1:500, Bioss Antibodies Inc., Woburn, Massachusetts, USA, bs-0465R), mouse anti-NfκB p50 (1:500, Santa Cruz Biotechnology, Dallas, TX, USA, sc-8414), rabbit anti-Akt1/PKB pS473 (1:200, Pleasanton, California, USA, E13454), rabbit anti-Fbp (1:1000, isolated, purified, and validated as described in^62^), mouse anti-Fbp (1:200, Santa Cruz Biotechnology, Dallas, TX, USA, sc-271799), rabbit anti-Tomm20 (1:1000, Merck KGaA, Darmstadt, Germany, HPA011562), mouse anti-phospho-Gsk3β (Ser9) (1:1000, Merck KGaA, Darmstadt, Germany, 05–643), mouse anti-Gfap (1:1000, Merck KGaA, Darmstadt, Germany, G3893), rabbit anti-Gfap (1:1000, Merck KGaA, Darmstadt, Germany, G926), rabbit anti-Mct2 (1:1000, Bioss Antibodies Inc., Woburn, Massachusetts, USA, Bs-3995R), mouse anti-Map2 (1:1000, Merck KGaA, Darmstadt, Germany, M4403). The primary antibodies were detected using appropriate fluorophore-labeled secondary antibodies: goat anti-rabbit-AlexaFluor488 (1:2000, ThermoFisher Scientific, Waltham, MA, USA, A11034), goat anti-mouse-AlexaFluor633 (1:2000, ThermoFisher Scientific, Waltham, MA, USA, A21050) and goat anti-mouse TRITC (1:1000, Merck KGaA, Darmstadt, Germany, T7782). Nuclei were counterstained with DAPI.

### ROS, [Ca^2+^] and mitochondria polarization assays

For intracellular ROS visualization, 5 μM CellROX Deep Red Reagent (ThermoFisher Scientific, Waltham, MA, USA, C10422) was used. The reagent was diluted in warm HBSS (ThermoFisher Scientific, Waltham, MA, USA, 14180046) and applied on astrocytic cultures for 30 min, 37 °C. Then, the cells were washed once with warm HBSS, fixed and microscopic images were taken immediately.

To determine mitochondrial ROS levels, the 500 nM MitoTracker Red CM-H_2_XRos (ThermoFisher Scientific, Waltham, MA, USA, M7513) was used according to the manufacturer’s instructions. Briefly, cells were washed two times with HBSS and incubated with the reagent for 30 min in the cell culture incubator. Then the cells were washed again with HBSS, fixed, and examined.

For the intracellular calcium observation, 2.5 μM Fluo-3/AM (ENZO Life Sciences, Farmingdale, New York, ALX-620-003-M001) was used. The reagent was dissolved in HBSS and applied on astrocytes for 30 min in room temperature (RT). This was followed by 20 min de-esterification of the reagent in the culture medium (in RT). To determine the contribution of extracellular calcium to the observed changes, astrocytes were incubated with the Ca^2+^-free medium for the time of de-esterification. Then the cells were washed briefly with HBSS and examined immediately, without fixation.

To determine mitochondrial calcium levels, 0.25 μM Rhod-2 AM (ThermoFisher Scientific, Waltham, MA, USA, R1244) was used. The experimental procedure was the same as for Fluo-3/AM, except for image acquisition, where excitation/emission wavelengths appropriate for Rhod-2 were used.

Mitochondrial membrane polarization was measured using the MitoPT JC-1 Assay (ImmunoChemistry Technologies, Davis, CA, USA, #924) according to the manufacturer’s instructions. 1 µM JC-1 reagent was dissolved in the assay buffer and applied on astrocytic cultures for 30 min, 37 °C. Immediately after the incubation unfixed astrocytes were observed in the confocal microscope. Mitochondria with a proper membrane polarization emitted light at 590 nm (red), depolarized mitochondria emitted light at 527 nm (green). The 590 nm/527 nm fluorescence intensity ratio was calculated. As the control, the mitochondrial membrane-depolarizing agent, 1 μM carbonyl cyanide 4-(trifluoromethoxy)phenylhydrazone (FCCP) (Merck KGaA, Darmstadt, Germany), was used.

### Fluorescence in situ hybridization (FISH)

FISH was performed according to^12^. The oligonucleotide for Fbp2 mRNA detection has the following sequence: (5′-[Cyanine3]GCACACAGCT GAGATACTCT TGCACATCCT CAGGGGAC-3′) and was synthesized by Merck KGaA, Darmstadt, Germany. Cells were fixed with 4% paraformaldehyde (PFA) in PBS, washed 2 times with PBS (5 min) and permeabilized with 0.3% Triton in PBS (15 min). It was followed by 10 min incubation in 0.1M TEA (triethanolamine) and 5 min incubation in 0.25% acetic anhydride in 0.1M TEA. After rinsing 3 times with PBS, 0.5 × SSC solution (20 × SSC: 3M NaCl, 0.3M sodium citrate) was added for 10 min and then 1 × SCC solution for 5 min. This was followed by a pre-hybridization in a moist chamber (50% formamide, 5 × SSC) in RT for 1 h. The composition of the pre-hybridization solution was: 50% formamide, 5 × Denhart's solution (100 × Denhart’s solution: 2% Ficoll-400, 2% BSA, 2% PVP), 5 mM EDTA, 4 × SSC. Hybridization was performed at 56 °C (i.e. the hybridization temperature of the Fbp2 probe) for about 18 h. The composition of the hybridization solution was: 50% formamide, 5 × Denhart's solution, 5 mM EDTA, 4 × SSC, 250 μg/mL tRNA, 500 μg/mL ssDNA, 2 μg probe.

### Confocal microscopy

The Olympus FV100 confocal microscope equipped with Plan Apo 60x/1.35 NA Oil and 40xUPlanSApo 40x/0.95 NA objectives was used for microscopic observations. Pixel size was 0.5 µm, exposure time 2 µs.

The ImageJ software^59^ was used for fluorescence quantification. The mean fluorescence intensity was measured from regions of interest (ROIs) and normalized to cell area. For protein-related fluorescence, ROIs delineating cell and nucleus boundaries were defined based on, respectively, Gfap immunostaining and DAPI fluorescence. To establish the nuclear/cytoplasmic ratio of a protein, the protein-associated fluorescence from the nucleus (DAPI) area was measured and expressed as a ratio to the fluorescence from the cytoplasmic (Gfap) area from the same cell. For Fbp2-mitochondria colocalization analysis the Manders’ overlap coefficient was used. The coefficient varies from 0 (no colocalization) to 1 (100% colocalization) and here, it was defined as the ratio of summed intensities of pixels from the mitochondria (Tomm20; magenta) channel for which the intensity in the Fbp2 (green) channel was above 0 to the total intensity in the mitochondrial channel^63^. In our experiments, the Manders’ overlap coefficient was normalized to control in different samples. After the normalization, 1 indicates overlap of the fluorescent signals in control conditions, and values above 1 indicate increasing and below 1 indicate decreasing overlap of these signals.

Each experiment was repeated at least in triplicate.

### Mitochondrial swelling measurement

Astrocytic mitochondria were isolated as described in^64^. The mitochondrial swelling measurement was conducted as reported previously^19^. Swelling of the organelles was identified as a decrease in light absorbance at 540 nm. The wild-type Fbp2 was expressed and purified as described in^22^ with minor modifications described in^62^. Mitochondrial swelling was induced by addition of 10 μM CaCl_2_ to 100 µg of isolated astrocytic mitochondria suspended in 500 μl of the swelling buffer (125 mM KCl, 10 mM HEPES, 0.5 mM MgCl_2_, 3 mM KH_2_PO_4_, pH 7.4). Mitochondrial swelling in this control conditions was considered as 100%. To test Fbp2 influence on swelling, the organelles (100 µg) were pre-incubated with 0.2 µM of wild-type Fbp2 for 2 min before adding to the swelling buffer. This was followed by 10 μM CaCl_2_ addition. To test the effect of NAD^+^, 0.2 µM Fbp2 was saturated with 100 µM NAD^+^ for 2 min and then the mixture was incubated for another 2 min with mitochondria and after that swelling was induced with 10 μM CaCl_2_. To assess the effect of NAD^+^ on mitochondrial volume changes, the organelles were incubated with 100 μM NAD^+^ for 2 min before the induction of swelling.

### Lactate measurement

The Lactate-Glo™ Assay (Promega Corporation, Madison, USA, J5021) was used to measure changes in lactate concentration in the culture medium, according to the manufacturer’s instruction. Briefly, 20,000 astrocytes per well were seeded on the 12-well plate. Two days later, the culture medium was changed and NEVs were added to one half of the wells. After 48 h of incubation with NEVs, the culture medium was collected, diluted 100 times, and added in 1:1 volume ratio to the reaction mixture. Basal luminescence of the fresh culture medium was considered as the background for lactate measurement of untreated astrocytes. For astrocytes treated with NEVs, the fresh medium with the addition of NEVs was considered as the background for luminescence measurements. Luminescence was recorded in Lumi luminometer (MicroDigital Co., Ltd., Gyeonggi-do, Korea).

### Western blot

Western blot of astrocytic homogenates was performed as described in^65^ using the following antibodies: mouse anti-Hif1α (1:500, Merck KGaA, Darmstadt, Germany, SAB5200017), rabbit anti-hexokinase type II (1:1000, Merck KGaA, Darmstadt, Germany, AB3279), rabbit anti-Pfkm (1:500, Merck KGaA, Darmstadt, Germany, HPA002117), rabbit anti-lactate dehydrogenase A (1:500, bio-techne, Toronto, Canada, NBP1-48336), rabbit anti-Pkm2 (isoform M1) (1:1800, Merck KGaA, Darmstadt, Germany, SAB4200094). For the loading control, the rabbit anti-actin primary antibody (1:10,000, Merck KGaA, Darmstadt, Germany, A2066) was used. Goat anti-rabbit peroxidase-conjugated antibody (1:40,000, Merck KGaA, Darmstadt, Germany, A0545) and goat anti-mouse peroxidase-conjugated antibody (1:30,000, Merck KGaA, Darmstadt, Germany, A9309) were used as secondary antibodies. The peroxidase reaction was visualized using 3,3′-diaminobenzidine (DAB) as the substrate. The densitometric analysis of protein bands was performed using the ImageJ software. The signal from a studied protein was normalized to the signal obtained for β-actin. For each analyzed protein the experiment was repeated in triplicate. Representative Western blots were chosen for the presentation of the results. The presented fold of change is based on three measurements.

### Kinetics

Inhibition of FBP2 by NAD^+^ was measured at pH 7.5, 37 °C using the glucose-6-phosphate isomerase–glucose-6-phosphate dehydrogenase coupled spectrophotometric assay as described previously^58^ with variable concentration of NAD^+^. The kinetic parameters were calculated using GraFit Version 4 software (Erithacus Software Limited, Surrey, UK). The data on the effect of the allosteric inhibition of FBP2 was fitted to the following equation:

$$\frac{v}{Vmax}=1-\frac{\left(Imax*{L}^{n}\right)}{IC{50}^{n}}+{L}^{n}$$

where v is the observed velocity at a specific concentration of allosteric effector—NAD^+^, Vmax is the activity in the absence of inhibitor, Imax is the maximal FBP2 inhibition caused by allosteric inhibitors, [L] is the concentration of NAD^+^, [n] is Hill coefficient and IC50 is the concentration of AMP/iFBP that causes 50% change in the maximal velocity.

### Data quantification, presentation, and statistical analysis

If not stated otherwise, all the experiments were performed in triplicate, i.e. there were 3 biological replicates (independent experiments using cell cultures derived from different batches of animals), and for each biological replicate there were 3 technical replicates (3 culture wells for each experimental condition) performed simultaneously. A biological replicate was considered to have been performed correctly and subjected to further analysis if there were no significant differences between its technical replicates under the same experimental conditions. Detailed information on the number of biological replicates, number of analyzed images or measurements for each condition, and statistical test results for all experiments are provided in Supplementary Table S1.

Fluorescence intensity was normalized to an appropriate control group so that the average value in control group was 1, and visualized with box plots (except the Manders’ coefficient). Plots show the results collected from all biological replicates. On the plots, center lines show the overall medians, box limits indicate the 25th and 75th percentiles, whiskers extend 1.5 times the interquartile range from the 25th and 75th percentiles, dots represent median value for each biological replicate. An individual measurement was the mean fluorescence intensity from the cells in one microscopic image. For statistical analyses, a nested ANOVA with two factors (biological replicate and condition) was used^66,67^. Biological replicate (random factor) refers to independently obtained primary cell cultures and is nested within a condition (fixed factor) that refers to a given treatment (e.g., control or addition of extracellular vesicels). Response variable is a protein level/colocalization coefficient defined by fluorescence intensity measurement/Manders’ coefficient in an immunodetection experiment. The experimental design is summarized in Supplementary Figure S6. For mitochondria swelling analysis (Fig. 4c) a repeated measures ANOVA was used. On the plots, presented p-values refer to post-hoc analysis (Tukey HSD test) of differences between different conditions. Cumulative distribution plots of normalized Manders’ coefficient show the results collected from all biological replicates, and two-sample Kolmogorov–Smirnov test was performed to assess whether two samples come from the same distribution. Results regarding the analysis of variance are presented in Supplementary Table S1. A probability of *p* < 0.05 was considered to be a significant difference. N refers to the total number of biological replicates performed. All statistical analyses were performed using R Statistical Software (v4.3.1; R Core Team 2023).

## Supplementary Information

Supplementary Information.

## Data Availability

Data generated during the study are available from the authors (please contact the corresponding author: agnieszka.gizak@uwr.edu.pl) upon reasonable request and with permission from the Narodowe Centrum Nauki (National Science Centre).
