# Author Correction: Neuronal extracellular vesicles influence the expression, degradation and oligomeric state of fructose 1,6-bisphosphatase 2 in astrocytes affecting their glycolytic capacity

**DOI:** 10.1038/s41598-024-75417-x

**Published:** 2024-10-14

**Authors:** Daria Hajka, Bartosz Budziak, Dariusz Rakus, Agnieszka Gizak

**Affiliations:** 1 Department of Molecular Physiology and Neurobiology, University of Wrocław, 50-335 Wrocław, Poland; 2 Present Address: Łukasiewicz Research Network - PORT Polish Center for Technology Development, 54-006 Wrocław, Poland

Correction to: *Scientific Reports *10.1038/s41598-024-71560-7, published online 09 September 2024

The Acknowledgements section in the original version of this Article was omitted. It now reads:

“Co-financed from the funds for scientific research and commercialization of their results of the University of Wrocław.”

The original Article has been corrected.

