# Supplementary material for: Neuronal extracellular vesicles influence the expression, degradation and oligomeric state of fructose 1,6-bisphosphatase 2 in astrocytes affecting their glycolytic capacity
Source: Sci Rep. 2024 Sep 9;14:20932. doi: 10.1038/s41598-024-71560-7 (PMC11385182; doi:10.1038/s41598-024-71560-7)
Supplement: Supplementary file 1 — Supplementary Information. [file 41598_2024_71560_MOESM1_ESM.docx]

**Supplementary Information**

**Neuronal extracellular vesicles influence the expression, degradation and oligomeric state of fructose 1,6-bisphosphatase 2 in astrocytes affecting their glycolytic capacity**

**Daria Hajka^1^, Bartosz Budziak^1^, Dariusz Rakus^1^, Agnieszka Gizak^1,*^**

**^1^** University of Wrocław, Department of Molecular Physiology and Neurobiology, Wrocław, 50-335, Poland

* agnieszka.gizak@uwr.edu.pl

The current address of Daria Hajka is: Łukasiewicz Research Network - PORT Polish Center for Technology Development, Wrocław, 54-006, Poland;


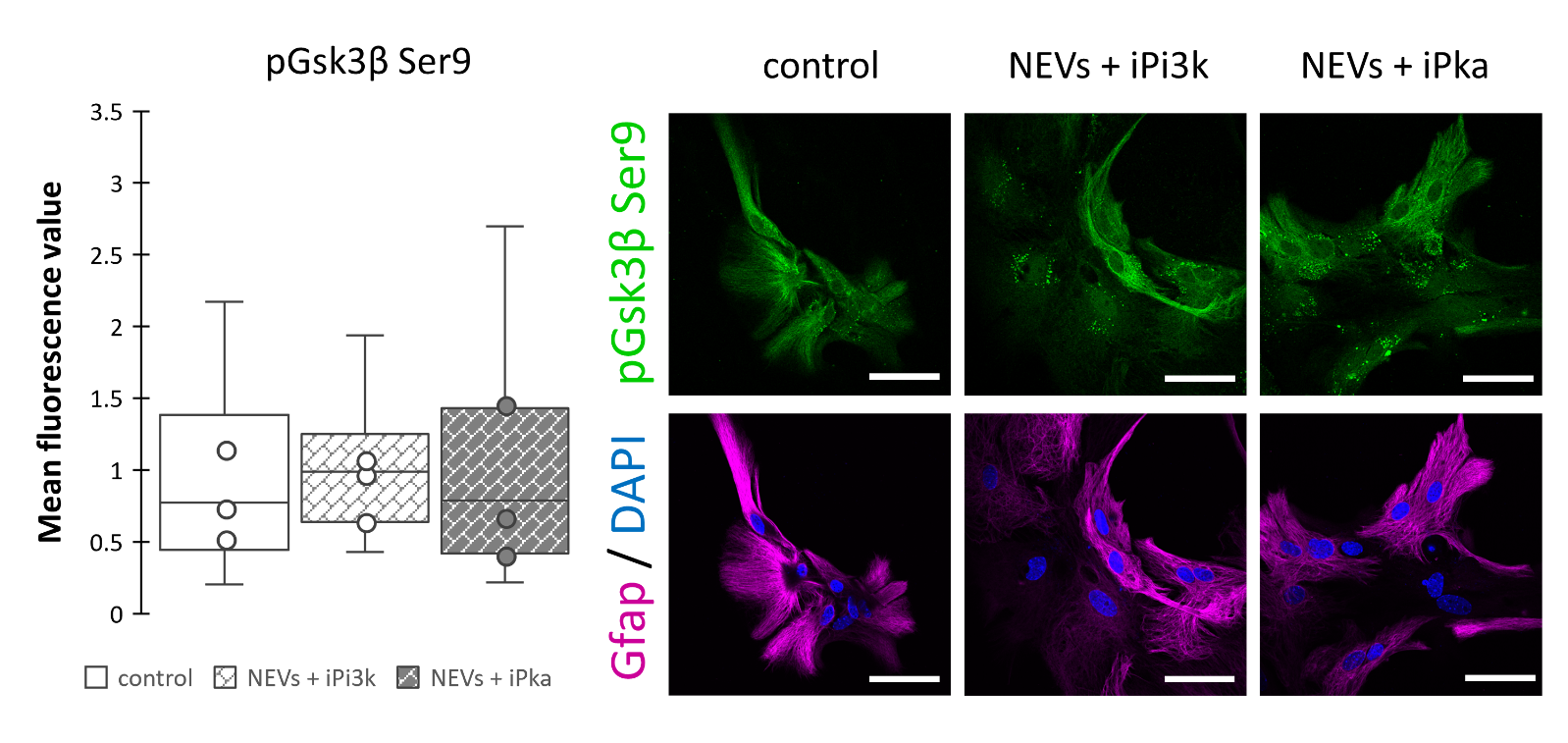
**Supplementary Figure S1. Inhibition of Pka and Pi3k kinases prevents NEVs from increasing the level of Gsk3β phosphorylation at Ser9**

Representative confocal images and quantification of the fluorescent signal related to antibodies directed against a given protein normalized to a respective control (untreated astrocytes). In a box plot, the horizontal line represents the overall median and dots represent median value for each biological replicate. The experiment was performed in triplicate (N = 3) . Astrocytes were incubated with neuronal extracellular vesicles (NEVs) in the presence of inhibitor of Pi3k (iPi3k; wortmannin) or inhibitor of Pka (iPka; KT5720). Bar=40 µm. Detailed information on the number of biological replicates, number of analyzed images or measurements for each condition, and statistical test results for all experiments are provided in Supplementary Table S1.


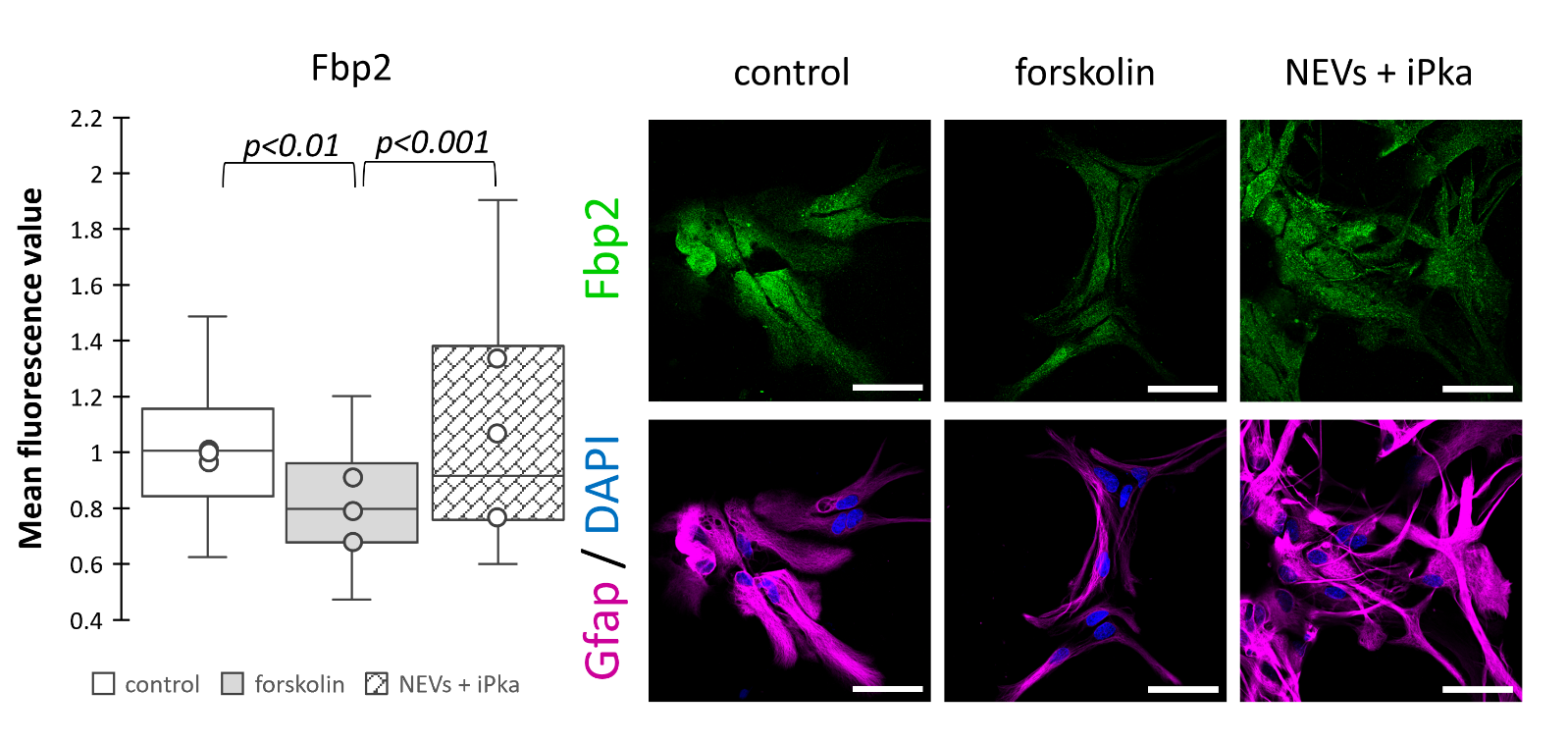
**Supplementary Figure S2. Inhibition of Pka prevents NEVs from reducing the amount of Fbp2 while activation of Pka reduce the amount of Fbp2**

Representative confocal images and quantification of the fluorescent signal related to antibodies directed against a given protein normalized to a respective control (untreated astrocytes). In a box plot, the horizontal line represents the overall median and dots represent median value for each biological replicate. The experiment was performed ~~at least~~ in triplicate (N = 3). Astrocytes were incubated with forskolin – activator of Pka or neuronal extracellular vesicles (NEVs) in the presence of Pka inhibitor (iPka; KT5720). Bar=40 µm. Detailed information on the number of biological replicates, analyzed images or measurements for each condition, and statistical test results for all experiments are provided in Supplementary Table S1.


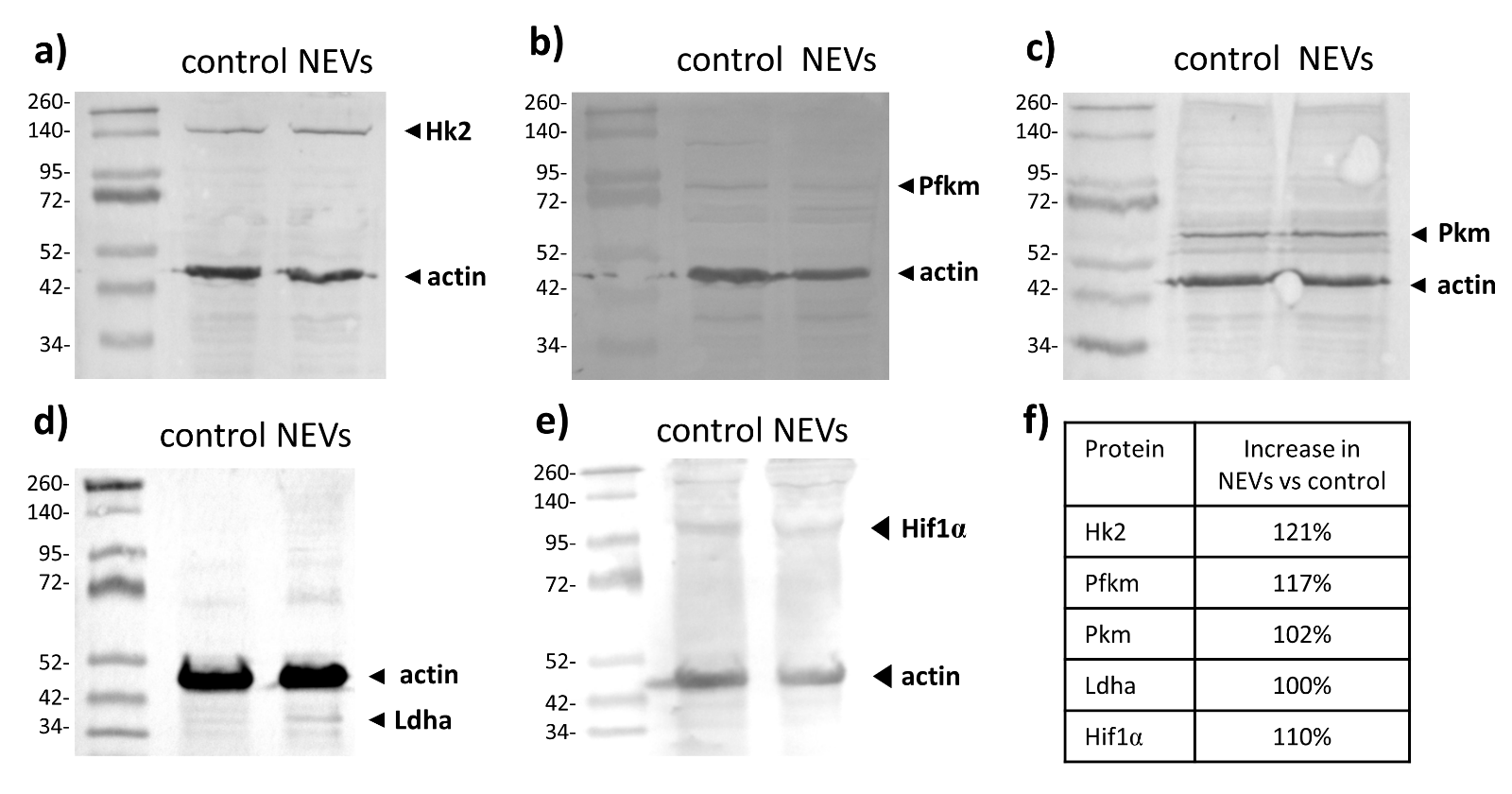


**Supplementary Figure S3. Neuronal extracellular vesicles increase glycolytic capacity of astrocytes elevating the expression of rate-limiting and regulatory proteins of glycolysis: hexokinase and phosphofructokinase, and stabilizing Hif1α**

A-E) Representative WB images and F) quantification of signal related to antibodies directed against a given protein. Each experiment was performed in triplicate (N = 3). NEVs – astrocytes treated with neuronal extracellular vesicles; Hk2 – hexokinase 2; Pfkm – phosphofructokinase M; Pkm - pyruvate kinase type M; Ldha - lactate dehydrogenase A; Hif1α – hypoxia-indusible factor 1α.


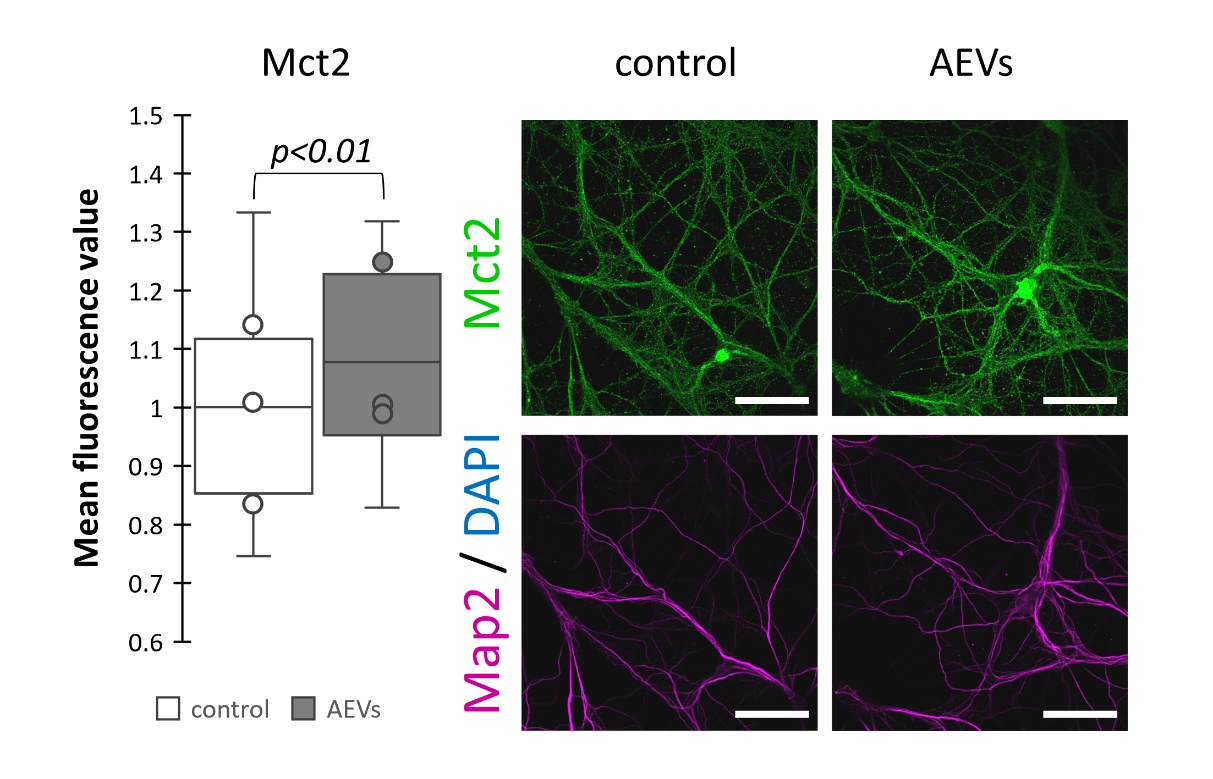


**Supplementary Figure S4. Astrocytic extracellular vesicles increase the amount of Mct2 protein in neurons**

Representative confocal images and quantification of the fluorescent signal related to antibodies directed against a given protein and normalized to a respective control (untreated neurons). In a box plot, the horizontal line represents the overall median and dots represent median value for each biological replicate. The experiment was performed in triplicate (N = 3). AEVs – neurons treated with astrocytic extracellular vesicles; Mct2 - monocarboxylate transporter 2; Map2 - microtubule-associated protein 2. Bar=40 µm. Detailed information on the number of biological replicates, analyzed images or measurements for each condition, and statistical test results for all experiments are provided in Supplementary Table S1.


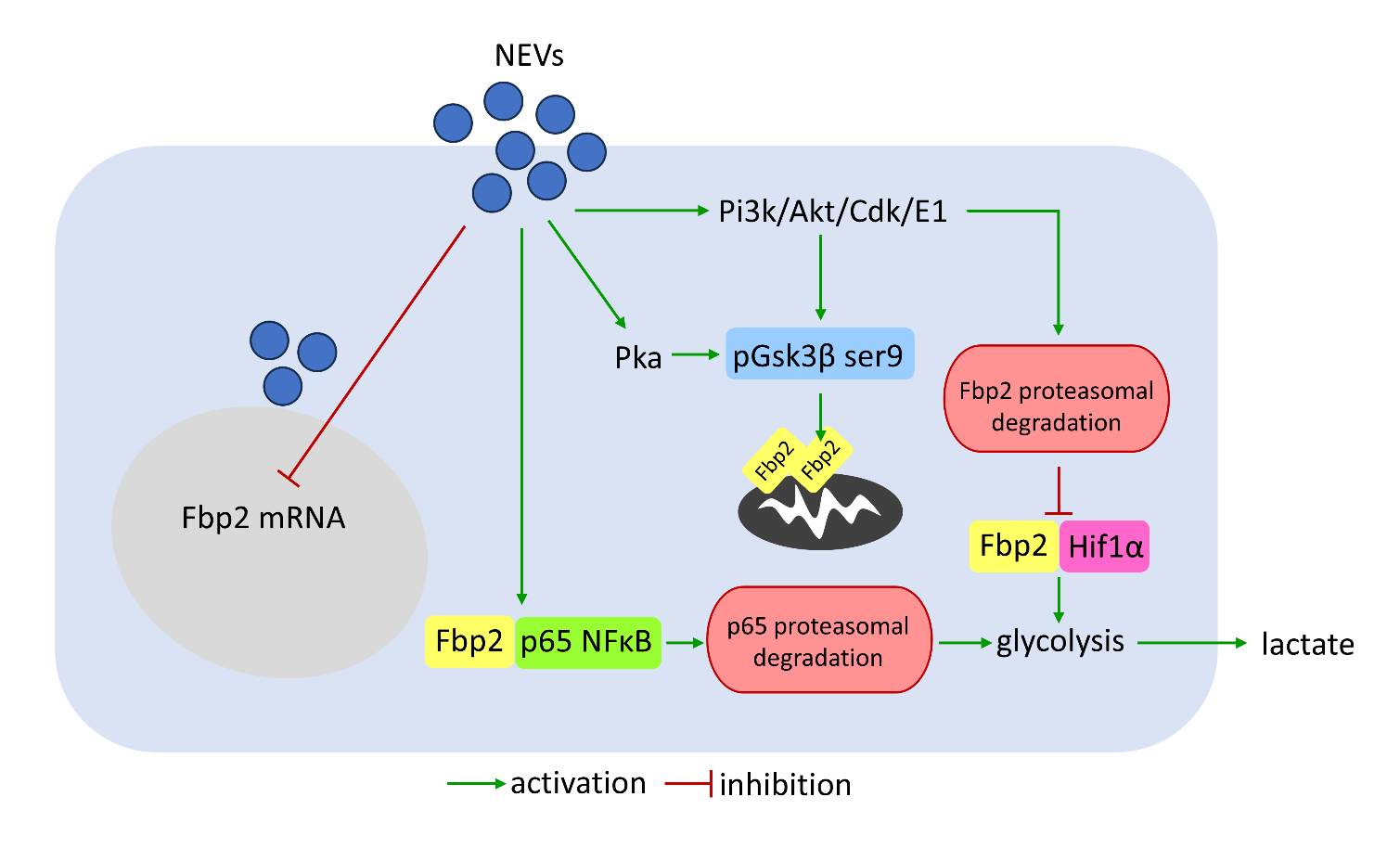


**Supplementary Figure S5. Summary of the NEVs-delivered cargo on Fbp2 in astrocytes**

The culture of astrocytes in the presence of NEVs results in a fast reduction of Fbp2 mRNA (in an unknown manner) and slower reduction of Fbp2 protein (by inducing its proteasomal degradation via Pi3k/Akt/Cdk/E1 pathway). This limits the Fbp2-Hif-1α interactions and increases glycolysis and lactate production. At the same time, increase in the inhibitory phosphorylation of Gsk3β by Pi3k/Akt and Pka results in translocation of Fbp2 dimers to mitochondria to protect their membrane potential against the effects of the NEVs-induced cellular [Ca^2+^] elevation. It is unclear whether (and how) the reduction of Fbp2 directly translates into the decrease of p65 Nf-κB.

Green pointed arrows – activation; red blunt arrows – inhibition; a question mark – an unknown mechanism.


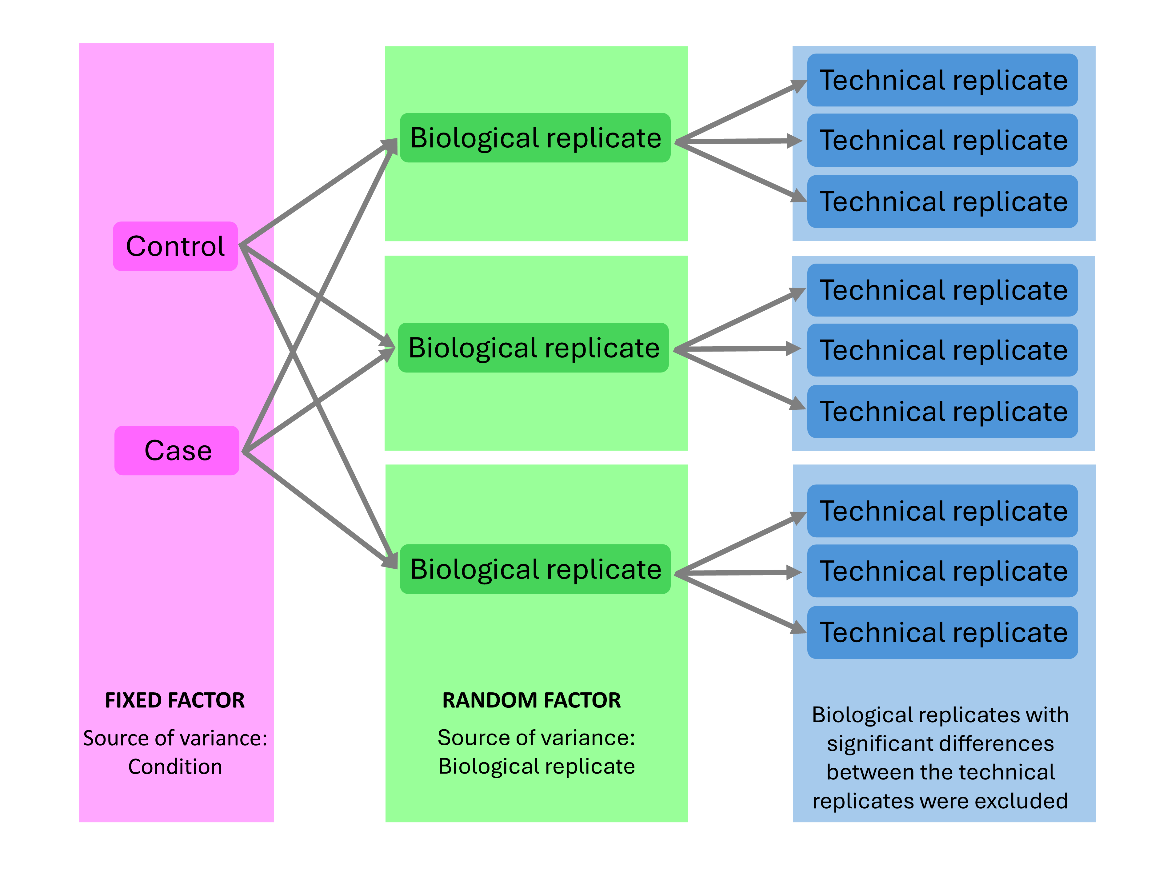


**Supplementary Figure S6. Experimental design**

For statistical analyses, a nested ANOVA with two factors (biological replicate and condition) was used. Biological replicate (random factor) refers to independently obtained primary cell cultures and is nested within a condition (fixed factor) that refers to a given treatment (e.g., control or addition of extracellular vesicels). Response variable is a protein level/colocalization coefficient defined by fluorescence intensity measurement/Manders’ coefficient in an immunodetection experiment.

**Supplementary Table S1. Detailed information on the number of biological replicates, analyzed images or measurements for each condition, and statistical test results**

Results of nested ANOVA or repeated measures ANOVA (Fig. 4c). For the nested ANOVA, two factors were (biological replicate and condition) taken into account. Presented p-values indicate the source of variance for each experiment, the corresponding post-hoc p-values are shown in each figure.

| **Figure** | **Number of biological replicates** | **Number of analyzed images or measurements for each condition** | **Nested ANOVA results** | |
| --- | --- | --- | --- | --- |
|  |  |  | **Source of variance** | **p-value** |
| Figure 1 | 3 | Control: 10, 10, 10 | Condition | p=1.11e-5 |
|  |  | NEVs 2h: 8, 8, 8 | Biological replicate | p=0.00185 |
|  |  | NEVs 48h: 10, 10, 10 |  |  |
| Figure 2a | 3 | Control: 10, 10, 10 | Condition | p=0.00738 |
|  |  | NEVs: 10, 9, 9 | Biological replicate | p=0.00103 |
| Figure 2b | 3 | Control: 10, 10, 10 | Condition | p=5.23e-6 |
|  |  | NEVs: 10, 10, 10 | Biological replicate | p=0.000168 |
| Figure 2c | 3 | Control: 10, 10, 10 | Condition | p=2e-16 |
|  |  | NEVs: 13, 13, 11 |  |  |
|  |  | NEVs + iPi3k: 10, 10, 10 | Biological replicate | p=0.503 |
|  |  | NEVs + iCdk: 10, 10, 10 |  |  |
| Figure 3a | 3 | Control: 21, 10, 13 | Condition | p=4.11e-5 |
|  |  | NEVs: 21, 10, 14 | Biological replicate | p=1.16e-8 |
| Figure 3b | 3 | Control: 12, 11, 12 | Condition | p=0.000258 |
|  |  | NEVs: 12, 11, 12 | Biological replicate | p=0.243782 |
| Figure 3c | 3 | Control: 15, 40, 18 | Condition | p=2e-16 |
|  |  | NEVs: 15, 16, 15 |  |  |
|  |  | NEVs + iFbp2: 15, 15, 15 | Biological replicate | p=0.996 |
|  |  | FCCP: 6, 6, 6 |  |  |
| Figure 3d | 3 | Control: 30, 30, 30 | Condition | p=2e-16 |
|  |  | NEVs: 30, 40, 15 | Biological replicate | p=0.00842 |
|  |  | NEVs + iFbp2: 13, 13, 14 |  |  |
| Figure 3e | 3 | Control: 15, 15, 30 | Condition | p=4.11e-6 |
|  |  | NEVs: 15, 15, 15 | Biological replicate | p=0.412 |
|  |  | NEVs + iFbp2: 13, 13, 14 |  |  |
| Figure 4a | 3 | Control: 23, 23, 24 | Condition | p=2e-16 |
|  |  | NEVs: 23, 23, 24 | Biological replicate | p=4.76e-7 |
|  |  | Control – [Ca^2+^]: 10, 10 ,10 | Condition | p=0.000773 |
|  |  | NEVs – [Ca^2+^]: 10, 10, 10 | Biological replicate | p=0.0512 |
| Figure 4b | 3 | Control: 20, 20, 20 | Condition | p=6.41e-6 |
|  |  | NEVs: 16, 16, 18 | Biological replicate | p=0.851 |
| Figure 4c | 3 | Control mitochondria: 1 | Condition | p=0.045264 |
|  |  | Mitochondria + iFbp2: 1 |  |  |
|  |  | Mitochondria + [Fbp2 + NAD^+^]: 1 | Time | p=3e-6 |
|  |  | Mitochondria + NAD^+^: 1 |  |  |
| Figure 5a | 3 | Control: 10, 10, 10 | Condition | p=4.7e-5 |
|  |  | NEVs: 10, 10, 10 | Biological replicate | p=0.412 |
| Figure 5b | 3 | Control: 20, 20, 20 | Condition | p=3.83e-10 |
|  |  | NEVs: 15, 15, 15 |  |  |
|  |  | NEVs + iPi3k: 10, 10, 10 | Biological replicate | p=2e-16 |
|  |  | Forskolin: 10, 10, 10 |  |  |
|  |  | NEVs + iPka: 10, 10, 10 |  |  |
| Figure 6a | 4 | Control: 15, 9, 13, 13 | Condition | p=0.00303 |
|  |  | NEVs: 15, 9, 13, 13 | Biological replicate | p=0.39478 |
| Figure 6b | 3 | Control: 7, 7, 6 | Condition | p=4.3e-12 |
|  |  | NEVs: 7, 7, 6 | Biological replicate | p=0.125 |
| Figure 6c | 3 | Control: 10, 10, 10 | Condition | p=0.70491 |
|  |  | NEVs: 10, 10, 10 | Biological replicate | p=0.00142 |
| Figure 7a | 3 | Control: 15, 10, 15 | Condition | P=0.01 |
|  |  | NEVs: 15, 10, 15 | Biological replicate | P=0.899 |
| Figure 7b | 4 | Control: 10, 15, 10, 10 | Condition | P=0.0011 |
|  |  | NEVs: 10, 15, 10, 10 | Biological replicate | p=0.1585 |
| Figure 7c | 3 | Control: 10, 15, 15 | Condition | p=0.792 |
|  |  | NEVs: 10, 15, 15 | Biological replicate | p=5.52e-6 |
| Figure 7d | 4 | Control: 15, 10, 15, 15 | Condition | p=0.4621 |
|  |  | NEVs: 15, 10, 15, 15 | Biological replicate | p=0.0183 |
| Figure 7e | 3 | Control: 15, 14, 15 | Condition | p=2.92e-5 |
|  |  | NEVs: 15, 14, 15 | Biological replicate | p=1.08e-11 |
| Figure 7f | 3 | Control: 15, 10, 10 | Condition | p=3.35e-7 |
|  |  | NEVs: 15, 10, 10 | Biological replicate | p=0.000396 |
| Figure 7g | 3 | Control: 9, 9, 9 | Condition | p=0.00422 |
|  |  | NEVs: 9, 9, 9 | Biological replicate | p=0.01376 |
| Supplementary Figure S1 | 3 | Control: 10, 10, 10 | Condition | p=0.99269 |
|  |  | NEVs + iPi3k: 10, 10, 10 | Biological repicate | p=0.00492 |
|  |  | NEVs + iPka: 10, 10, 10 |  |  |
| Supplementary Figure S2 | 3 | Control: 13, 13, 14 | Condition | p=5.49e-6 |
|  |  | Forskolin: 16, 16, 18 | Biological repicate | p=5.35e-5 |
|  |  | NEVs + iPka: 10, 10, 10 |  |  |
| Supplementary Figure S4 | 3 | Control: 10, 10, 10 | Condition | p=0.00179 |
|  |  | AEVs: 10, 10, 10 | Biological replicate | p=5.35e-9 |
